# Cellular homeostasis of *N*-acetylneuraminic acid and non-canonical sialic acids is mediated by human *N*-acetylneuraminate lyase

**DOI:** 10.1093/glycob/cwag038

**Published:** 2026-05-18

**Authors:** Sjanie Huang, Iris Harmsen, Moritz Rahm, Takfarinas Kentache, Clara D M van Karnebeek, Afitz Da Silva, Alexey V Pshezhetsky, Emile Van Schaftingen, Alejandro Garanto, Dirk J Lefeber

**Affiliations:** Department of Neurology, Donders Institute for Brain, Cognition and Behaviour, Radboud University Medical Center, Geert Grooteplein 10, 6525 GA, Nijmegen, The Netherlands; Department of Human Genetics, Radboud University Medical Center, Geert Grooteplein 10, 6525 GA, Nijmegen, The Netherlands; Department of Human Genetics, Radboud University Medical Center, Geert Grooteplein 10, 6525 GA, Nijmegen, The Netherlands; European Research Institute for the Biology of Ageing, University of Groningen, University Medical Center Groningen, Antonius Deusinglaan 1, 9713 AV, Groningen, The Netherlands; Department of Neurology, Donders Institute for Brain, Cognition and Behaviour, Radboud University Medical Center, Geert Grooteplein 10, 6525 GA, Nijmegen, The Netherlands; Department of Human Genetics, Radboud University Medical Center, Geert Grooteplein 10, 6525 GA, Nijmegen, The Netherlands; Laboratory of Physiological Chemistry, De Duve Institute, UCLouvain, Avenue Hippocrate 75, 1200 Brussels, Belgium; United for Metabolic Diseases, Meibergdreef 9, 1105 AZ, Amsterdam, The Netherlands; Emma Center for Personalized Medicine, Departments of Pediatrics and Human Genetics, Amsterdam UMC, Meibergdreef 9, 1105 AZ, Amsterdam, The Netherlands; Department of Pediatrics, Centre Hospitalier Universitaire Sainte-Justine Research Center, University of Montréal, 3175 chemin de la Côte-Sainte-Catherine, Montréal QC H3T 1C5, Canada; Department of Pediatrics, Centre Hospitalier Universitaire Sainte-Justine Research Center, University of Montréal, 3175 chemin de la Côte-Sainte-Catherine, Montréal QC H3T 1C5, Canada; Department of Anatomy and Cell Biology, McGill University, 3640 University Street, Montréal QC H3A 0C7, Canada; Laboratory of Physiological Chemistry, De Duve Institute, UCLouvain, Avenue Hippocrate 75, 1200 Brussels, Belgium; Department of Human Genetics, Radboud University Medical Center, Geert Grooteplein 10, 6525 GA, Nijmegen, The Netherlands; Department of Pediatrics, Amalia Children's Hospital, Radboud University Medical Center, Geert Grooteplein 10, 6525 GA, Nijmegen, The Netherlands; Department of Neurology, Donders Institute for Brain, Cognition and Behaviour, Radboud University Medical Center, Geert Grooteplein 10, 6525 GA, Nijmegen, The Netherlands; Department of Human Genetics, Radboud University Medical Center, Geert Grooteplein 10, 6525 GA, Nijmegen, The Netherlands; United for Metabolic Diseases, Meibergdreef 9, 1105 AZ, Amsterdam, The Netherlands

**Keywords:** enzyme function, metabolism, *N*-acetylneuraminate lyase, neuraminic acid, sialic acid

## Abstract

Sialic acids are important for cellular communication, with *N*-acetylneuraminic acid (Neu5Ac) being the canonical form of sialic acid in humans. Presence of non-canonical sialic acids, derived from dietary intake or as metabolic side product, has been linked to immune disorders and cancer. As homeostasis of different sialic acids remains poorly understood in humans, we studied the role of *N*-acetylneuraminate lyase (NPL) in their catabolism. In vitro expression of NPL in different biological systems revealed broad substrate specificity towards sialic acids and related 2-keto-3-deoxy metabolites. In agreement with the broad substrate specificity, NPL-deficient red blood cells accumulated Neu5Ac and 3-deoxy-d-*glycero*-d-*galacto*-nonulosonic acid (KDN). Interestingly, endogenous levels of non-canonical sialic acids, including *N*-glycolylneuraminic acid (Neu5Gc) and KDN, were depleted in HEK293T cells upon NPL overexpression, while Neu5Ac and CMP-Neu5Ac levels remained stable. This was further confirmed by supplementation with different sialic acids. Detailed analysis of sugar phosphate intermediates of the hexosamine and sialic acid biosynthesis pathways showed strongly elevated ManNAc-6P (*N*-acetyl-d-mannosamine 6-phosphate) and Neu5Ac-9P, indicating efficient recycling of ManNAc to increase de novo Neu5Ac biosynthesis. However, this recycling was not efficient for Neu5Gc and KDN. While GlcNAc-6P (*N*-acetyl-d-glucosamine 6-phosphate) levels were slightly elevated, no evidence was found for further metabolism towards GlcN-6P (glucosamine 6-phosphate) and energy production via glycolysis as shown for bacterial neuraminate lyases. In conclusion, human NPL catabolizes a broad range of sialic acids. However, depending on the cellular context, NPL contributes to a net cellular reduction in non-canonical sialic acids, such as KDN, due to a lack of efficient recycling.

## Introduction

The ancient family of nonulosonic acids consists of nine-carbon monosaccharides, of which the 3-deoxy forms are referred to as sialic acids (Lewis et al. 2023). Different forms of sialic acids can be found across the domains of life, primarily in the deuterostome lineage of the animal kingdom and in certain groups of bacteria (Angata and Varki 2002). Sialic acids are involved in glycosylation of proteins and lipids, and are also present as polysialic acids in humans and bacterial species, such as *Neisseria meningitidis* (Bhattacharjee et al. 1975). Positioned as the outermost sugar moiety in glycoconjugates, sialic acids are crucial for cellular communication via recognition by receptors on host cells or pathogens (Varki 2007). The canonical human sialic acid, *N*-acetylneuraminic acid (Neu5Ac), is involved in multiple biological processes, such as brain development, immune responses, and reproduction (Varki 2008; Wang 2009; Schnaar et al. 2014). Its essential role is further emphasized by the severe human phenotypes that result from genetic defects in Neu5Ac biosynthesis, such as epilepsy and neurodegeneration in NANS deficiency (MIM 610442), and severe myopathy in GNE deficiency (MIM 605820) (van Karnebeek et al. 2017; Mashangva et al. 2024).

In the majority of vertebrates, Neu5Ac and Neu5Gc (*N*-glycolylneuraminic acid) are the predominant sialic acid forms incorporated into glycoconjugates (Varki 2009). However, Neu5Ac is the dominant form in human glycoconjugates, while Neu5Gc is only present in limited amounts (Tangvoranuntakul et al. 2003). Lack of Neu5Gc occurred during human evolution when the gene coding for the enzyme CMP-Neu5Ac hydroxylase (CMAH) became inactivated, which is required for de novo synthesis of CMP-Neu5Gc (Irie et al. 1998). As a consequence, Neu5Gc is recognized as non-human by the immune system when incorporated from the diet into glycoconjugates (Tangvoranuntakul et al. 2003; Bardor et al. 2005; Padler-Karavani et al. 2008). The presence of Neu5Gc-containing glycans in human tissues has been shown to induce inflammation, atherosclerosis, and cancer progression (Hedlund et al. 2008; Samraj et al. 2015; Kawanishi et al. 2019). Circulating antibodies have been detected in humans that are able to recognize glycans containing Neu5Gc and KDN (3-deoxy-d-*glycero*-d-*galacto*-nonulosonic acid) (Padler-Karavani et al. 2008; Kawanishi et al. 2021; Saha et al. 2021). KDN is another non-canonical form of sialic acid that is produced as side product by the enzyme *N*-acetylneuraminic acid synthase (NANS) by using mannose 6-phosphate as substrate instead of the canonical *N-*acetyl-d-mannosamine 6-phosphate (ManNAc-6P) (Lawrence et al. 2000). Although multiple studies have shown the presence of free and bound KDN in various human cell lines and human material, little is known about its function in humans (Inoue et al. 1998; Inoue et al. 2006; Chen et al. 2014; Wang et al. 2023; Wang et al. 2024). Previously, elevated levels of free KDN have been associated with cancer and end-stage renal disease (Inoue et al. 1998; Kawanishi et al. 2021), and KDN was hypothesized to act as a buffer to prevent potential harmful effects of mannose 6-phosphate (Kawanishi et al. 2021). The current knowledge about the homeostasis of different forms of sialic acids in humans is limited, however, it can be anticipated that their levels should be tightly controlled due to aforementioned negative health effects.

**Figure 1:**
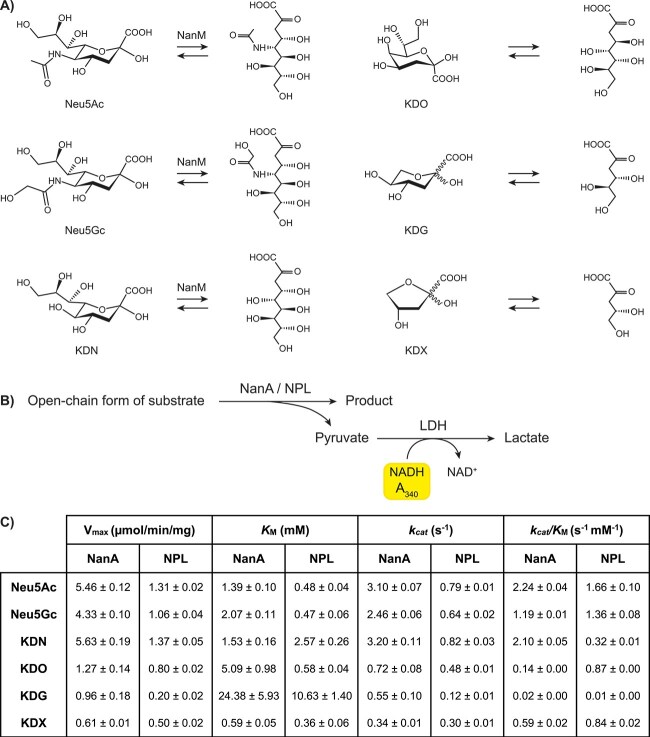
Substrate specificity of *E. Coli* NanA and human NPL. (A) Chemical structures of the sialic acids Neu5Ac, Neu5Gc, and KDN, and the structurally similar molecules KDO, KDX, and KDG are shown in their cyclic and open-chain forms. The interconversion of the cyclic and open-chain forms of Neu5Ac, Neu5Gc, and KDN is catalysed by the mutarotase NanM, which was therefore added during the enzyme assay with these substrates to facilitate ring opening, while KDG and KDX are already largely in their linear form. Addition of NanM was not required for assays with KDO, KDG, and KDX. (B) Chemical reaction of the NADH-coupled enzyme assay to determine lyase activity. Generated pyruvate was converted to lactate by lactate dehydrogenase (LDH), and NADH consumption was monitored via absorbance at 340 nm. (C) Kinetic parameters of cleavage of substrates by *E. Coli* NanA and human NPL determined by an NADH-coupled enzyme assay. Data are represented as the mean ± SEM, *n* = 3.

*N*-acetylneuraminate lyase (NPL) is the enzyme required for catabolism of sialic acids. NPL activity has been detected in human red blood cells (Bulai et al. 2002), however, the exact role of NPL in human physiology remains unclear. *NanA* (*N*-acetylneuraminate lyase), the bacterial homolog of *NPL*, is involved in energy production and virulence via the use of sialic acids as a carbon source (Vimr et al. 2000; Hopkins et al. 2013; Vo et al. 2022). Patients have been reported with a defect in NPL function, resulting in elevated Neu5Ac levels in urine and red blood cells, but normal levels in fibroblasts (Wen et al. 2018). Clinically, patients have a neuromuscular disease presenting symptoms such as muscle weakness, exercise intolerance, and dilated cardiomyopathy (Wen et al. 2018). Zebrafish and mouse models of NPL deficiency have recapitulated the human phenotype and confirmed the importance of NPL in muscle development and function (Wen et al. 2018; Da Silva et al. 2023). Previously, Neu5Ac and Neu5Gc, but not KDN, have been identified as substrates of human NPL (Bulai et al. 2002; Wen et al. 2018), while NanA from *Escherichia coli* has broad substrate specificity. These include the sialic acids Neu5Ac, Neu5Gc, and KDN, and the structurally similar sugar KDO (3-deoxy-d-*manno*-octulosonic acid) (Fig. 1A) (Uchida et al. 1984; Wada et al. 2003; Kentache et al. 2021).

In this study, we observed a broad in vitro substrate specificity of human NPL towards Neu5Ac, Neu5Gc, KDN, and the structurally related molecules KDO and KDX (2-keto-3-deoxy-d-xylonate). In contrast, cellular levels of Neu5Ac and CMP-Neu5Ac were stable upon NPL overexpression due to their increased de novo biosynthesis, while all other sialic acids assessed were depleted and effective recycling was absent. Our results suggest that NPL may have a key role in balancing levels of Neu5Ac and undesired sialic acids in response to the metabolic status of the cell.

## Results

### In vitro assessment of human NPL enzyme activity shows broad substrate specificity towards sialic acids and structurally similar molecules

To study the substrate specificity of human NPL, we used recombinantly expressed NPL and compared substrate specificity with *E. coli* NanA. The enzyme kinetic parameters were determined for several substrates using an NADH-coupled NPL enzyme assay in which pyruvate production was measured indirectly (Fig. 1B). We studied lyase activity towards three sialic acids (Neu5Ac, Neu5Gc, and KDN) and three structurally similar 2-keto-3-deoxy molecules: I) KDO, which is an octulosonic acid and can be found in lipopolysaccharides of Gram-negative bacteria and in the cell wall of plants (Heath and Ghalambor 1963; York et al. 1985); II) the hexulose 2-keto-3-deoxy-d-gluconate (KDG), which is an intermediate of hexuronate metabolism in bacteria, such as *E. coli* (Pouyssegur and Stoeber 1974); and III) the pentulose KDX, which is an intermediate in the Weimberg pathway of certain bacteria (Weimberg 1961). Recently, it was demonstrated that NPL and NanA act on the open forms of Neu5Ac and KDN (Kentache et al. 2021), rather than the alpha cyclic forms as previously reported (Ooi et al. 2000). In this enzyme assay, the mutarotase NanM was therefore added to speed up the conversion of the cyclic form to the open-chain form for selected substrates: Neu5Ac, Neu5Gc, and KDN (Fig. 1A). Since KDG and KDX are largely present in their linear form, addition of NanM was not required to facilitate ring opening (Kentache et al. 2021).

The kinetic parameters obtained for NanA and NPL for the different substrates are listed in Fig. 1C. NanA was efficient in cleaving Neu5Ac, KDN, and Neu5Gc as represented by the catalytic efficiencies (*k_cat_/K*_M_) of 2.24, 2.10, and 1.19 mM^−1^ s^−1^, respectively (Figs. 1C and S1A). NanA enzymatic activities towards KDO and KDX were also detected, but with lower catalytic efficiencies, while KDG was not a substrate of NanA (Figs. 1C and S1A). These results are in line with previous studies showing the flexibility of NanA towards a broad range of substrates (Uchida et al. 1984; Wada et al. 2003; Kentache et al. 2021). Regarding KDG, another aldolase has been identified in *E. coli* (KdgA) acting on the phosphorylated form of KDG, namely 2-keto-3-deoxy-6-phosphogluconate (KDPG) (Blanco et al. 1983).

The preferred substrate for human NPL was Neu5Ac with a *k_cat_/K*_M_ value of 1.66 mM^−1^ s^−1^, followed by Neu5Gc with 1.36 mM^−1^ s^−1^ (Figs. 1C and S1B). Neu5Ac and Neu5Gc have previously been reported experimentally as substrates of NPL (Bulai et al. 2002; Wen et al. 2018; Cheng et al. 2022). In addition to these two known substrates of NPL, we here showed that NPL is able to cleave KDN, KDO, and KDX as well, but not KDG (Figs. 1C and S1B). In the previous study, no NPL activity could be detected towards KDN (Wen et al. 2018). This could be explained by their choice of enzyme assay, which was based on ManNAc detection, while KDN cleavage yields mannose as product. Our assay circumvents this limitation, since it relies on indirect detection of pyruvate formation, which occurs in all tested enzyme reactions (Fig. 1B). Furthermore, our findings confirmed the previously predicted binding and cleavage of KDO by NPL based on homology modelling (Chu et al. 2009). Interestingly, the catalytic efficiency of NPL towards KDN (0.32 mM^−1^ s^−1^) was the lowest of all substrates (Figs. 1C and S1B). This was mostly because of the 5-fold higher *K*_M_ for KDN in comparison to Neu5Ac as substrate, indicating a lower affinity of NPL for KDN. This is in clear contrast with the substrate preference of NanA, were Neu5Ac and KDN have very similar and high catalytic efficiencies. On the other hand, NPL has a higher affinity towards KDO compared to NanA (Figs. 1C and S1). Overall, both NanA and NPL were able to cleave Neu5Ac, Neu5Gc, KDN, KDO, and KDX. Some key differences were observed between NanA and NPL in the obtained kinetic parameters, mainly regarding the catalytic efficiencies for KDN and KDO.

### Cellular assessment of sialic acid levels suggests a preference of NPL for catabolism of KDN and Neu5Gc over Neu5Ac

**Figure 2:**
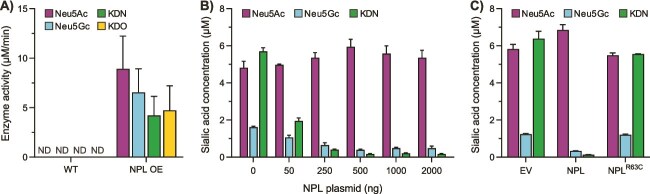
Assessment of enzyme activity and sialic acid concentrations in HEK293T cells transfected with NPL plasmid. (A) NPL enzymatic activity could not be detected in WT HEK293T cell lysates, but it was observed in NPL OE cell lysates with Neu5Ac, Neu5Gc, KDN, and KDO as substrates as determined in the NADH-coupled enzyme assay. Data are represented as the mean of two assays with two biological replicates per assay ± SD. ND (not detected) indicates that enzyme activity was below the detection threshold. (B) the concentration of Neu5Gc and KDN were decreased in a dose-dependent manner with increasing NPL plasmid doses (0–2000 ng), while Neu5Ac concentration remained unchanged. The sialic acid concentrations (μM) were determined in transfected HEK293T cells by LC–MS/MS using ^13^C-Neu5Ac as internal standard. (C) the sialic acid levels were compared in HEK293T cells transfected with an empty vector (EV) as negative control, WT NPL plasmid (NPL), or NPL plasmid carrying the patient p.Arg63Cys variant (NPL^R63C^). Unlike the WT NPL plasmid, overexpression of NPL^R63C^ did not decrease concentrations of, Neu5Gc and KDN. (B-C) data are represented as the mean of three biological replicates ± SD.

To further study the function of human NPL in a cellular model with active metabolism, an NPL overexpression (OE) model was established. We transiently overexpressed human NPL in human embryonic kidney 293 T (HEK293T) cells, as confirmed by western blot (Fig. S2A). In view of their potential occurrence in humans, either as product of endogenous metabolism (Neu5Ac and KDN), food metabolism (Neu5Gc), or breakdown of bacteria (KDO), we selected Neu5Ac, Neu5Gc, KDN, and KDO as substrates for cellular studies. First, NPL activity was detected towards all substrates tested upon NPL overexpression (Fig. 2A), showing that human NPL has a broad substrate specificity independent of the expression system used for its production. The absence of NPL activity in WT cells is likely explained by a very low abundance of endogenous NPL in HEK293T cells in combination with the relatively low sensitivity of the assay. Next, we transfected HEK293T cells with increasing doses of NPL plasmid (0–2000 ng), which resulted in a gradual increase of NPL protein expression (Fig. S3), and analysed the cellular levels of the different sialic acids and KDO by ion-paring reverse phase liquid chromatography–tandem mass spectrometry (LC–MS/MS) (Fig. 2B). Neu5Ac and KDN were the most abundant sialic acids in HEK293T cells, while KDO was not detected at all, which was expected since KDO is mainly found in plants and bacteria (Heath and Ghalambor 1963; York et al. 1985). The presence of the non-human sialic acid Neu5Gc was likely originating from foetal bovine serum (FBS) in the culture medium. With increasing NPL overexpression, the concentrations of Neu5Gc and KDN were decreasing in a dose-dependent manner (Fig. 2B). Surprisingly, Neu5Ac levels remained stable, irrespective of NPL overexpression levels. When HEK293T cells were transfected with an NPL plasmid carrying the patient p.Arg63Cys variant (NPL^R63C^), we did not observe a decrease in Neu5Gc and KDN levels, further confirming that these effects were due to the catalytic activity of NPL (Fig. 2C).

To determine the contribution of endogenous NPL activity in wild-type cells, we performed genome editing by CRISPR/Cas9 to generate an *NPL* knockout (KO) model in HEK293T cells. Two NPL KO clones were selected, each generated with a different guide RNA (sgRNA) targeting exon 5 (Table S1). The insertion of knock-out mutations in the *NPL* gene were verified by Sanger sequencing, and off-target effects were not detected at the predicted sites (Figs. S2C and S4). NPL KO cells showed reduced *NPL* mRNA expression compared to WT cells and NPL protein was absent in western blot (Fig. S2B and S2D). Similar to WT HEK293T cells, NPL enzyme activity was not detected in NPL KO cells for all tested substrates: Neu5Ac, Neu5Gc, KDN, and KDO (Fig. 2A). In addition, sialic acid levels in NPL KO cells were comparable to WT cells (Fig. S2E). These results indicate that WT HEK293T cells have low endogenous expression levels of NPL. Possibly, these NPL expression levels are not sufficient for the formation of tetramers, which may be required for enzymatic activity (Devenish and Gerrard 2009). The low abundance and activity of endogenous NPL in HEK293T cells renders them a suitable model to study NPL protein function.

### Sialic acid supplementation reveals stable cellular levels of Neu5Ac and CMP-Neu5Ac upon NPL overexpression

**Figure 3:**
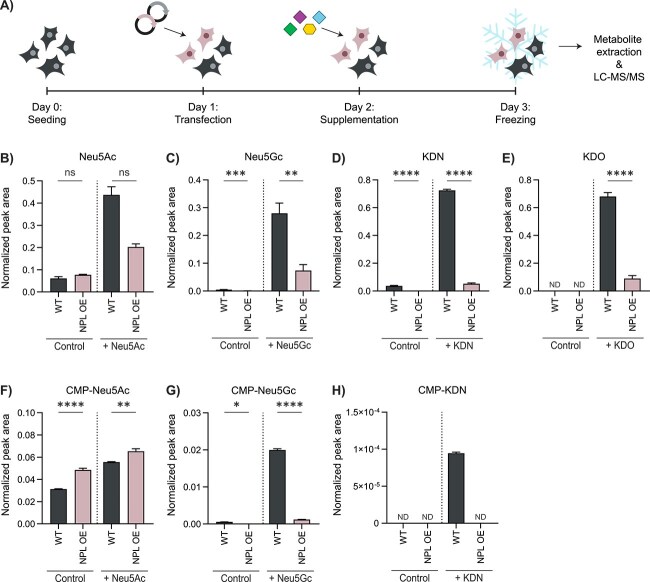
Sialic acid levels and their CMP-derivatives in WT and NPL OE HEK293T cells. A) Timeline of the in vitro experiment in HEK293T cell models. Cells were transfected with an empty vector (WT) or an *NPL* OE vector 24 h after seeding. On day 2, cells were supplemented with 1 mM Neu5Ac, Neu5Gc, KDN, or KDO, followed by freezing and metabolite extraction for LC–MS/MS. (B-H) normalized peak areas of (B) Neu5Ac, (C) Neu5Gc, (D) KDN, (E) KDO, (F) CMP-Neu5Ac, (G) CMP-Neu5Gc, and (H) CMP-KDN in the HEK293T NPL OE cell model cultured for 24 h in medium without supplementation (control) or with supplementation of 1 mM Neu5Ac, Neu5Gc, KDN, or KDO, respectively. Data are represented as the mean of three biological replicates ± SD. Statistical analyses were performed using (B) a Kolmogorov–Smirnov test or (C-H) an unpaired two-tailed *t-*test (^*^P < 0.05, ^**^P < 0.01, ^***^P < 0.001, ^****^P < 0.0001, ns: *P* > 0.05). ND indicates that the metabolite level in the sample was below the limit of detection.

To further confirm the differential cellular effects of NPL on different sialic acids, HEK293T cells were supplemented with 1 mM Neu5Ac, Neu5Gc, KDN, or KDO for 24 h and intracellular metabolites were analysed by LC–MS/MS (Fig. 3A). With this targeted metabolomics approach, different types of sialic acids could be detected, as well as polar metabolites from connected pathways (Dataset S1). Regarding the sialic acids, the levels of Neu5Ac, Neu5Gc, KDN, and KDO clearly increased in the WT and NPL KO cell lines upon supplementation (Figs. 3B-E and S5A-D). When NPL was overexpressed, the levels of Neu5Gc, KDN, and KDO were significantly reduced, indicating their catabolism by NPL (Fig. 3C-E). In contrast, Neu5Ac was not significantly decreased upon NPL overexpression and supplementation with Neu5Ac (Fig. 3B).

We next studied the levels of the corresponding nucleotide sugar products of the reaction catalysed by CMP-sialic acid synthetase (CMAS) that act as precursors for sialylation: CMP-Neu5Ac, CMP-Neu5Gc, CMP-KDN, and CMP-KDO (Figs. 3F-H, S5E-G, S6A-B, and Dataset S2). Since commercial standards were not available for CMP-Neu5Gc and CMP-KDN, these were synthesized. Synthetic CMP-Neu5Gc and CMP-KDN were prepared by incubation of the corresponding sialic acids and cytidine-5′-triphosphate (CTP) with CMAS from *N. meningitidis* group B. The products were confirmed by LC–MS/MS based on predicted multiple reaction monitoring (MRM) transitions related to CMP-Neu5Ac (Fig. S7 and Table S2). In WT and NPL KO cells, supplementation with the respective sialic acid substrate resulted in a minor increase of CMP-Neu5Ac (Figs. 3F and S5E), but a clear increase of CMP-Neu5Gc (Figs. 3G and S5F) and CMP-KDN (Figs. 3H and S5G). Presence of CMP-KDO was not detected in any of the samples, using the predicted transitions. This is in line with previous findings in which no activity was detected for human CMAS with KDO as substrate (Mertsalov et al. 2016). Under control conditions, CMP-Neu5Gc levels significantly declined with increased NPL overexpression (Fig. S6A). Although feeding with Neu5Gc slightly increased CMP-Neu5Gc levels in NPL OE cells, the levels remained significantly lower than in supplemented WT cells (Fig. 3G). The reduced CMP-Neu5Gc levels and absence of CMP-KDN in NPL OE cells implies that most Neu5Gc and KDN were efficiently broken down by NPL before it could be converted by CMAS. On the contrary, a minimal elevation of the CMP-Neu5Ac levels was detected in NPL OE cells compared to WT cells for both conditions (Figs. 3F and S6B). The observation that the effect on Neu5Ac and CMP-Neu5Ac was limited in the NPL OE model, combined with the similar enzymatic activity of NPL towards Neu5Ac, Neu5Gc, and KDN in the in vitro enzyme assay, suggests a compensatory mechanism to maintain Neu5Ac and CMP-Neu5Ac levels in the cell.

### Recycling of released ManNAc results in elevated de novo Neu5Ac biosynthesis to maintain Neu5Ac levels

**Figure 4:**
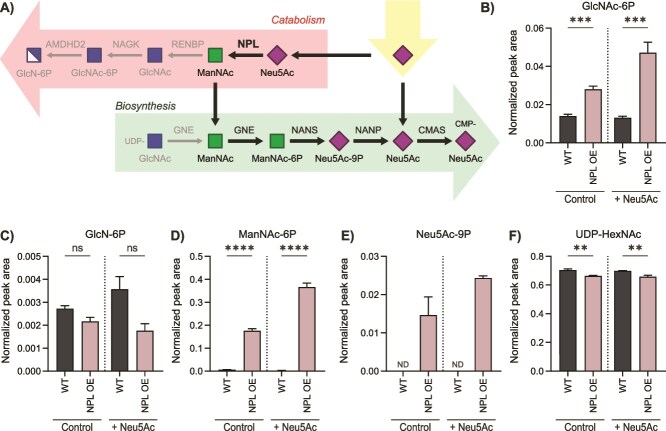
Elevated Neu5Ac biosynthesis upon cleavage of Neu5Ac into ManNAc and pyruvate by NPL. (A) Metabolic pathway of Neu5Ac catabolism and biosynthesis. The black arrows depict the proposed mechanism in NPL OE HEK293T cells in which increased de novo Neu5Ac biosynthesis is observed upon NPL-mediated Neu5Ac breakdown. Monosaccharide symbols follow the SNFG (symbol nomenclature for Glycans) system (Varki et al. 2015) (B-F) normalized peak areas of (B) GlcNAc-6P, (C) GlcN-6P, (D) ManNAc-6P, (E) Neu5Ac-9P, and (F) UDP-HexNAc in the HEK293T NPL OE cell model after 24 h in culture medium without supplementation (control) or with supplementation of 1 mM Neu5Ac. Data are represented as the mean of three biological replicates ± SD. Statistical analyses were performed using (A) a Kolmogorov–Smirnov test or (B, C, E) an unpaired two-tailed *t-*test (^**^P < 0.01, ^***^P < 0.001, ^****^P < 0.0001, ns: *P* > 0.05).

To understand the mechanism underlying the maintained Neu5Ac levels, we studied its downstream metabolites upon Neu5Ac catabolism (Figs. 4, S5H-K, S6C-E, and Datasets S1–2). In the first step, Neu5Ac is broken down by NPL, which yields ManNAc and pyruvate (Fig. 4A). ManNAc can be further converted to *N*-acetyl-d-glucosamine (GlcNAc) by *N*-acetyl-d-glucosamine 2-epimerase (RENBP) (Luchansky et al. 2003), followed by phosphorylation to GlcNAc-6P by *N*-acetyl-d-glucosamine kinase (NAGK) (Gindzienski et al. 1974). When cells were supplemented with 1 mM Neu5Ac for 24 h, indeed a 3.6-fold increase of GlcNAc-6P was observed in the NPL OE cell model (Fig. 4B). Next, GlcNAc-6P can be deacetylated to glucosamine 6-phosphate (GlcN-6P) by *N*-acetylglucosamine 6-phosphate deacetylase (AMDHD2) (Bergfeld et al. 2012). However, GlcN-6P appeared to be slightly decreased, but not significantly, in the NPL OE cell model upon feeding with Neu5Ac (Fig. 4C). These results demonstrate that catabolism of Neu5Ac by NPL increases levels of GlcNAc-6P, but not GlcN-6P.

Alternatively, ManNAc can be phosphorylated to ManNAc-6P by the bifunctional enzyme UDP-*N*-acetylglucosamine 2-epimerase/*N*-acetylmannosamine kinase (GNE). Thereby, ManNAc could be reused for Neu5Ac biosynthesis (Fig. 4A). ManNAc-6P levels were significantly increased by 28-fold in the NPL OE model, which was even more prominent after Neu5Ac supplementation with a 148-fold increase (Fig. 4D). The next step in Neu5Ac biosynthesis is the conversion of ManNAc-6P to Neu5Ac-9P by NANS. Indeed, Neu5Ac-9P was detected in the NPL OE model, while it was below the limit of detection in WT and NPL KO models (Fig. 4E). UDP-GlcNAc, the substrate for GNE epimerase activity, was measured as part of the UDP-*N*-acetyl-hexosamine (UDP-HexNAc) pool and was slightly decreased by 5.8% in NPL OE compared to WT for both control and supplemented conditions (Fig. 4F). The minimal change in UDP-GlcNAc levels could be explained by the fact that ManNAc enters the pathway after UDP-GlcNAc. Furthermore, a dose–response effect was observed between increasing ManNAc-6P and Neu5Ac-9P levels, and increasing NPL overexpression levels, while UDP-GlcNAc was not affected (Fig. S6C-E). Taken together, these results indicate that ManNAc is efficiently recycled via the de novo Neu5Ac biosynthesis pathway, thereby boosting de novo biosynthesis and sustaining the Neu5Ac and CMP-Neu5Ac levels (Fig. 4A).

Recycling of the non-canonical sialic acids KDN and Neu5Gc was less efficient. In analogy to Neu5Ac and Neu5Gc catabolism, KDN catabolism likely results in mannose, which could be recycled via mannose 6-phosphate, and converted to KDN-9P by NANS (Fig. S8A) (Lawrence et al. 2000). However, we did not observe any increase of mannose 6-phosphate in NPL OE cells upon KDN feeding (Dataset S1) and KDN-9P was not detected at all. To study the fate of Neu5Gc (Fig. S8B), we programmed predicted MRM transitions for HexNGc-6P (*N*-glycolylhexosamine 6-phosphate) isomers, and observed two minor peaks representing ManNGc-6P (*N*-glycolylmannosamine 6-phosphate) and GlcNGc-6P (*N*-glycolylglucosamine 6-phosphate) in NPL OE cells, which were increased upon Neu5Gc supplementation (Fig. S9 and Table S3). However, Neu5Gc-9P was not detected, indicating that Neu5Gc was not efficiently recycled under the conditions studied (Fig. S8B).

**Figure 5:**
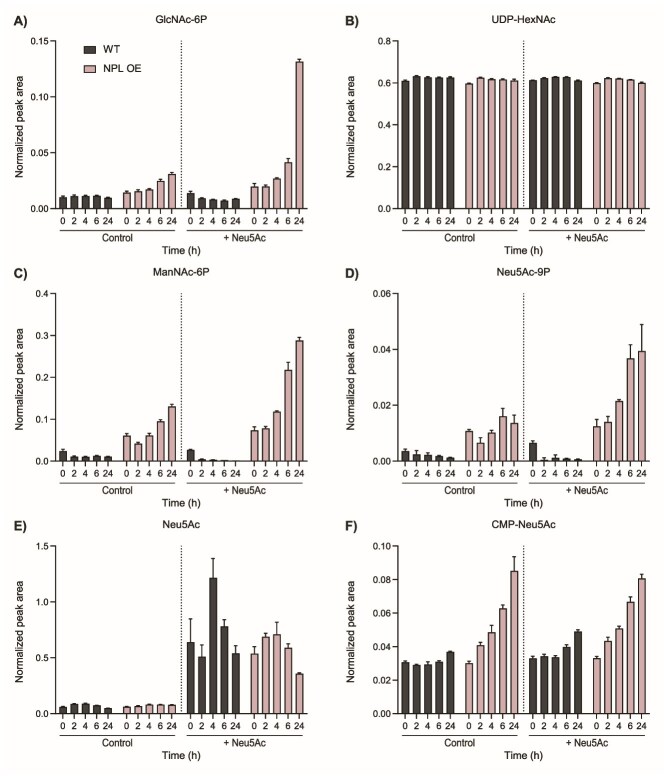
Gradual increase of Neu5Ac biosynthesis over time upon NPL overexpression. (A-F) normalized peak areas of (A) GlcNAc-6P, (B) UDP-HexNAc, (C) ManNAc-6P, (D) Neu5Ac-9P, (E) Neu5Ac, and (F) CMP-Neu5Ac in WT and NPL OE cells after 0, 2, 4, 6, and 24 h in culture medium without supplementation (control) or with supplementation of 1 mM Neu5Ac. Data are represented as the mean of three biological replicates ± SD.

To gain insight into the time course of ManNAc metabolism, we monitored metabolite levels over time in WT and NPL OE cells (Datasets S3 and S4). In agreement with previous experiment, GlcN-6P and UDP-HexNAc levels were similar in WT and NPL OE (Figs. 5B and S10). Both GlcNAc-6P and ManNAc-6P showed an increase from 4 h upon feeding with 1 mM Neu5Ac with a slightly slower response of GlcNAc-6P (Figs. 5A and 5C). This implies that the abundantly produced ManNAc was primarily metabolized via the kinase activity of GNE to form ManNAc-6P, while additional metabolism likely occurred via epimerization by RENBP and subsequent phosphorylation to GlcNAc-6P by kinase NAGK (Fig. 4A). Furthermore, ManNAc-6P levels were already elevated in the NPL OE cells at 0 h (Fig. 5C), which is explained because cells were transfected 24 h before Neu5Ac was supplemented (t = 0 h). Therefore, NPL was already actively metabolizing endogenous Neu5Ac before start of the supplementation. Neu5Ac-9P formation followed the same increasing trend as ManNAc-6P (Fig. 5D). Neu5Ac was observed to increase immediately intracellularly upon minutes after the feeding and then stabilized in the WT or decreased slightly in the NPL OE model (Fig. 5E). CMP-Neu5Ac levels increased gradually during the 24 h (Fig. 5F). These results show that ManNAc obtained from NPL-mediated catabolism is rapidly metabolized by downstream enzymes and thereby refuels the Neu5Ac biosynthesis pathway.

### KDN levels are elevated in NPL-deficient red blood cells

**Figure 6:**
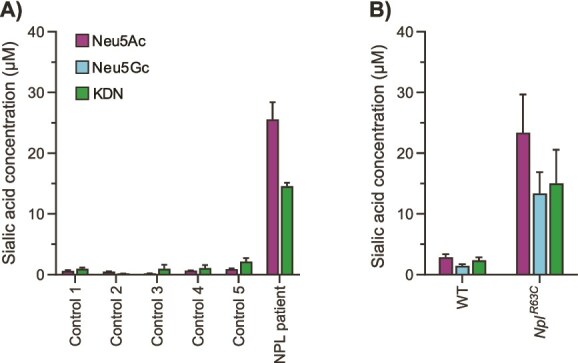
Accumulation of free sialic acids in NPL-deficient red blood cells. (A) Sialic acid concentration of Neu5Ac and KDN in red blood cells of five healthy controls and one NPL patient quantified by LC–MS/MS. Neu5Gc was not detected. (B) Sialic acid concentration of Neu5Ac, Neu5Gc, and KDN in red blood cells of WT and NPL-deficient (*Npl^R63C^*) mice. Data are represented as the mean of three technical replicates ± SD.

The results from enzyme activity assessments and the HEK293T NPL OE model showed that human NPL has a broad substrate specificity towards sialic acids. Our previous study demonstrated that patients with NPL deficiency had elevated levels of Neu5Ac in red blood cells, likely reflecting the usually high activity of NPL in healthy red blood cells (Wen et al. 2018). In addition, a second type of sialic acid, KDN, has been detected in human red blood cells (Inoue et al. 1998). We therefore remeasured previously obtained red blood cells derived from an NPL patient and identified an accumulation of both Neu5Ac and KDN (Fig. 6A), which is in line with our observations that human NPL is able to metabolize those sialic acids. Neu5Gc was not detected in human red blood cells. To confirm this further, we studied red blood cells of the previously published NPL-deficient mouse model (*Npl^R63C^*) (Da Silva et al. 2023), and observed elevation of both Neu5Ac and Neu5Gc, the canonical sialic acids in mice (Fig. 6B). In agreement with the findings in human NPL-deficient red blood cells, we also detected an increased KDN concentration in NPL-deficient mouse red blood cells.

## Discussion

Using different biological systems for the expression of NPL, we showed that human NPL is able to catabolize a broad range of sialic acids and structurally similar molecules. In agreement, NPL deficiency in humans and mice resulted in the accumulation of both canonical and non-canonical sialic acids. In a human cellular model, NPL overexpression resulted in a net depletion of KDN and Neu5Gc, while levels of Neu5Ac and CMP-Neu5Ac remained stable. Mechanistic analysis of intermediate metabolites indicated that ManNAc, produced by NPL activity, re-entered Neu5Ac biosynthesis to maintain cellular Neu5Ac levels, while there was a lack of efficient recycling for non-canonical sialic acid molecules.

*N*-acetylneuraminate pyruvate lyases comprise a group of tetrameric enzymes, which are composed of subunits with a (β/α)_8_-barrel (Izard et al. 1994). The most well studied group are the bacterial *N*-acetylneuraminate pyruvate lyases, which are able to convert a wide range of substrates. The *E. coli* NanA active enzyme is a tetramer, but is inactive as a dimer (Devenish and Gerrard 2009). Bacterial sialic acid aldolases have especially become useful in catalysing the reverse reaction for chemoenzymatic synthesis of sialic acids and their derivatives (Lv et al. 2017). These enzymes are able to accept different substrates, including various analogues, showing their substrate flexibility (Moons et al. 2019). Consistent with that, this study showed that human NPL has a broad substrate specificity as well. Interestingly, NanA has a higher affinity for KDN as the substrate than NPL does. Aside from Neu5Ac and Neu5Gc, *E. coli* is able to utilize KDN as sole carbon and energy source (Hopkins et al. 2013). It is yet to be determined how differences in kinetic parameters could be explained from a biological point of view.

Currently, little is known about the physiological relevance of sialic acid catabolism by NPL in humans. Based on literature and our novel data, two diverging hypotheses can be proposed: I) sialic acid catabolism is important for energy production, and II) NPL is required to reduce the levels of non-canonical sialic acids to prevent undesired biological effects, such as their effect on other metabolic pathways or their incorporation into newly synthesized glycans.

In several bacterial species, sialic acids are broken down by sialic acid aldolases to be used as carbon, nitrogen, or energy source (Vimr et al. 2004; Hopkins et al. 2013; Haines-Menges et al. 2015; McDonald et al. 2016). Being able to use host sialic acids is especially beneficial for pathogenic bacteria (Almagro-Moreno and Boyd 2009; Vo et al. 2022; Liang et al. 2023). Whether energy production could be a function of human sialic acid catabolism remains to be elucidated. Previous research has proposed a role for dietary sialic acid from milk oligosaccharides as nutrients in newborns (Nohle and Schauer 1984; Duncan et al. 2009; Sprenger and Duncan 2012). However, more recent studies argue that the majority of ingested sialic acid is quickly excreted via the urine and mainly has a prebiotic role (Galuska et al. 2020; Coker et al. 2021). The hypothesized role of sialic acid for energy production could be obtained through either one of the cleavage products, which are pyruvate and ManNAc. Pyruvate can enter the citric acid cycle after its conversion to acetyl-CoA, while ManNAc first has to be converted in multiple steps to fructose 6-phosphate before entering glycolysis. However, intermediate metabolites could also flow into other pathways. For example, GlcN-6P and GlcNAc-6P could enter into the hexosamine biosynthesis pathway for the production of UDP-GlcNAc, which can be used for O-GlcNAcylation or other glycosylation processes. In our NPL overexpressing cell model, we observed elevated GlcNAc-6P levels, but did not observe an effect on GlcN-6P or fructose 6-phosphate levels. In pancreatic cancer cells, NPL did not contribute to pyruvate generation either (Yu et al. 2019). Our findings do not clearly support the hypothesis of an existing flux by Neu5Ac catabolism from GlcNAc-6P towards glycolysis in the used HEK293T cell model.

It is important to note that our findings from a HEK293T cell model cannot be simply extrapolated to other cell types or tissues. Similar analyses in induced pluripotent stem cells along with differentiation into specific tissue types could prove a valuable human model system to gain further insights into NPL-related metabolism. Furthermore, the directionality of cellular metabolism may be impacted by the used culturing conditions, which was a nutrient-rich culture medium in our case, supporting anabolism. Other pathways may be preferred in different cellular systems or culture conditions, for example in more catabolic situations. The effect of NPL expression on cellular metabolism will also depend on the levels and cross-talk of multiple metabolic enzyme systems, such as NANS, involved in KDN production, and mannose and mannose 6-phosphate levels as precursors of KDN. Additional factors should be considered, including enzymes involved in synthesis of CMP-KDN, potential KDN incorporation into glycans, and KDN release and salvage via the lysosomal pathway. In this respect, overproduction of NPL may disturb this metabolic balance, even though our NPL dosing experiment suggests similar specificity in KDN catabolism at lower NPL concentrations.

As a second hypothesis we propose that human NPL has a role in eliminating non-canonical sialic acids to prevent their incorporation into glycans or in mitigating their possible negative effects on other metabolic pathways potentially causing detrimental health effects. In particular, several studies demonstrated the presence of Neu5Gc-containing glycans in human tissues (Tangvoranuntakul et al. 2003). Auto-antibodies have been detected in humans recognizing both Neu5Gc- and KDN-containing glycans, suggesting that these residues are recognized as non-self antigens (Padler-Karavani et al. 2008; Kawanishi et al. 2021; Saha et al. 2021), leading to chronic inflammation, also referred to as “xenosialitis” (Dhar et al. 2019). Furthermore, elevated levels of conjugated Neu5Gc have been found in disease states such as cancer and atherosclerosis (Hedlund et al. 2008; Samraj et al. 2015; Kawanishi et al. 2019). Interestingly, the absence of Neu5Gc in the vertebrate brain implies its possible harmful effects on the central nervous system (Davies and Varki 2015; Naito-Matsui et al. 2017). KDN incorporation into glycoconjugates is less well studied, and reports about occurrence of conjugated KDN in humans are rare (Kawanishi et al. 2021; Wang et al. 2023; Wang et al. 2024). KDN seems to be more abundant in various tumour cell lines (Inoue et al. 1998; Go et al. 2007). Noteworthy, one study showed that KDN incorporation into oligo- and polysialic acid chains terminated their elongation (Angata et al. 1994). In addition, Neu5Gc in polysialic acid is more resistant to removal by sialidases (Davies et al. 2012). Therefore, it is possible that control of non-canonical sialic acids may best be exerted on the free sialic acid level. Before sialic acids can be incorporated into glycans, they first have to be activated by CMAS to obtain nucleotide sugar donors. However, human CMAS is less efficient in activating KDN than Neu5Ac or Neu5Gc (Lawrence et al. 2001; Mertsalov et al. 2016). Thereby, the combination of lower activity of CMAS and catabolism by NPL may direct KDN for catabolism in contrast to the formation of CMP-KDN. Our study did not support a recycling towards mannose 6-phosphate and KDN-9P, however, mannose may be reduced to mannitol for secretion, similar to the production of galactitol in galactosemia, in which galactose accumulates (Jakobs et al. 1995). Alternatively, excess of KDN could potentially be directly excreted via the urine (Galuska et al. 2020; Kawanishi et al. 2021). Finally, KDN may exert negative effects on other intracellular metabolic pathways by itself. This is for instance seen with 4-phosphoerythronate and 2-phospho-L-lactate, which are toxic metabolic side products of glycolysis and inhibit other enzymatic reactions (Collard et al. 2016).

Past research in human and animal models has shown that NPL deficiency leads to muscle weakness and delayed muscle regeneration after injury (Wen et al. 2018; Da Silva et al. 2023). The skeletal muscle is an organ with a high energy demand. Therefore, lack of NPL activity may disturb energy metabolism as described in the first hypothesis, which may explain muscle symptoms observed in NPL myopathy patients. On the other hand, accumulation of Neu5Ac is characteristic in NPL myopathy patient urine and red blood cells (Wen et al. 2018). This study shows that KDN also accumulates in red blood cells from an NPL myopathy patient and from NPL-deficient mice. As mentioned in the second hypothesis, accumulation of KDN might be undesired and cause disease. However, additional research is needed to understand the biological function and possible toxicity of free KDN. Previously, it has been proposed that KDN may act as a buffer system to prevent mannose 6-phosphate and fructose 6-phosphate accumulation (Kawanishi et al. 2021). NPL may also further metabolize formed KDN in such conditions towards other metabolic pathways to prevent accumulation of potentially toxic mannose 6-phosphate. Furthermore, it is needed to elucidate to what extent KDN accumulates in different tissues and body fluids of NPL myopathy patients, and whether concentrations are reached that promote KDN incorporation into glycans. In mice, incorporation of the appropriate sialic acid type at the right timing is essential for muscle differentiation (Go et al. 2017; Go et al. 2022). A shift towards higher sialylated N-glycans was observed in NPL-deficient mouse muscles by glycomics, in particular bearing higher levels of Neu5Gc (Da Silva et al. 2023). Furthermore, studies have been performed in bacteria where *NPL* gene homologs were knocked out, which demonstrated an increase of multiple types of free sialic acid and their incorporation into glycans (Vimr et al. 2000; Saha et al. 2021). Through identification of novel substrates for human NPL, our study offers new insights into the pathological mechanism of NPL myopathy and provide targets for treatment of patients affected by this rare disorder of sialic metabolism.

Overall, our study revealed the broad substrate specificity of human NPL and led us to the hypothesis of controlling non-canonical sialic acids as the biological function of NPL. It remains to be elucidated what the physiological function of free sialic acids is, and whether it is necessary for human health to regulate (non-)human sialic acids through NPL. As our study indicated the importance of the cellular context to understand the function of human NPL, additional research should further explore this in relevant tissue-specific models.

## Materials and methods

### Expression and purification of recombinant enzymes in *E. Coli*

NanA, NanM, and NPL were expressed as previously described (Kentache et al. 2021), and additional experimental details including the used bacterial strains and cloning of human *NPL* in the pET22b(+) vector are described in the Supplementary material.

### HEK293T cell culture

HEK293T cells (CRL-3216; ATCC) were cultured in high glucose (4.5 g/L) Dulbecco’s Modified Eagle Medium (DMEM) with pyruvate (21969035; Gibco) supplemented with 10% (v/v) FBS (10270106; Gibco), 2 mM GlutaMAX (Gibco), 100 U/mL penicillin and 100 mg/mL streptomycin (Gibco). To detach and passage the cells, 0.25% trypsin (Gibco) was used. Cells were maintained at 37 °C and 5% CO_2_.

### *NPL* overexpression vector generation and transfection of HEK293T cells

In order to transiently overexpress human *NPL* in HEK293T cells, an *NPL* expression vector was generated using the Gateway cloning system (Thermo Scientific) (Katzen 2007). First, human *NPL* (isoform NPL-203, transcript ID: ENST00000367553.6) was amplified using *NPL* forward and reverse primers with *att*B tails (hNPL_2; Table S1) by polymerase chain reaction (PCR) using Phusion™ High-Fidelity DNA Polymerase (Thermo Scientific). Complementary DNA (cDNA) from HEK293T cells was used as the DNA template. Subsequently, the purified product was cloned into a pDONR™201 vector (Invitrogen) through the BP reaction to generate the entry vector. Homemade DH5α competent cells were transformed with the entry vector to isolate plasmid from selected colonies. Finally, through an LR reaction *NPL* was cloned into the expression vector pcDNA3. All positive clones were validated by restriction analysis and Sanger sequencing. The construction of DYK-tagged NPL and NPL^R63C^ plasmids was described previously (Wen et al. 2018). HEK293T cells were seeded in 6-well plates for transient overexpression of NPL. Cells were transfected with the *NPL* expression vector (0–2 μg) using FuGENE® HD transfection reagent (Promega) and Opti-MEM™ (Gibco) following the instructions of the manufacturer. A FuGENE:plasmid ratio of 3:1 (3 μl FuGENE® HD transfection reagent for 1 μg of plasmid) was used. Cells were harvested 72 h post-transfection for protein detection by western blot.

### Generation of HEK293T *NPL* knockout cell lines

Two sgRNAs (Table S1) targeting *NPL* exon 5 were designed using the CRISPOR tool (http://crispor.tefor.net/) (Concordet and Haeussler 2018). Each sgRNA was then cloned into a PX458 plasmid backbone which contains the green fluorescent protein (GFP) as a reporter following the protocol published elsewhere (Ran et al. 2013). Two micrograms of each plasmid obtained was used to transfect HEK293T cells grown in a 6-well with FuGENE® HD transfection reagent according to the manufacturer’s instructions using the 3:1 ratio. Expression of GFP allowed the detection and sorting of transfected cells as single cells into a 96-well plate by a Gallios flow cytometer (Beckman Coulter). Clones were grown and screened for successful *NPL* gene editing by Sanger sequencing using primers flanking the CRISPR/Cas9 target site (Table S1). Once NPL KO clones were identified, CRISPR/Cas9 off-target effects were excluded by Sanger sequencing. The predicted off-target sites were selected based on CRISPOR scores considering the number of mismatches and the presence of an NGG protospacer adjacent motif (PAM). Amplification of the off-target site and Sanger sequencing were performed using the primers listed in Table S1. NPL KO clones were validated by quantitative real-time PCR (qRT-PCR) and western blotting (Supplementary material).

### Measurement of NanA and NPL activities

The enzyme activity of purified NanA and NPL was determined based on NADH disappearance measured at 340 nm using a spectrophotometer (Beckman Coulter DU-800 series). The reaction mixture contained 50 mM HEPES (pH 8.0), 10 mM KCl, 0.5 mM dithiothreitol (DTT), 1 mM MgCl_2_, 0.5 mg/mL bovine serum albumin (BSA), and 140 μM NADH at 37 °C. Several substrates of the sialic acid family were tested (Neu5Ac, Neu5Gc, KDN, KDO, KDX, and KDG; Sigma-Aldrich) at indicated concentrations. The coupling enzyme lactate dehydrogenase (LDH; EC 1.1.1.27; Roche; 5.0 μM) and NanM (0.5 μM) were added to the mixture. The reaction was started by adding NanA or NPL at 1 μM in a final volume of 600 μL. Presented data are V_max_, turnover number *k_cat_*, K_M_, and *k_cat_*/K_M_, which were determined by nonlinear curve fitting with GraphPad Prism (v10.1.2) assuming the reaction followed Michaelis–Menten kinetics.

NPL activity was measured in HEK293T cell lysates diluted to a total protein amount of 50 μg. The protein concentration in cell lysates was quantified using the Pierce™ BCA protein assay kit (Thermo Scientific) following manufacturer’s instructions. The same reaction mixture was used as described above, except for a higher NADH concentration of 900 μM. In a 96-well plate, substrates were added at a concentration of 20 mM to the reaction mixture, while Milli-Q (MQ) water was added in case of the blank. In this assay, only LDH was added as coupling enzyme. The reaction was started by adding 25 μl of cell lysate to obtain a final volume of 120 μL. Decrease in NADH absorbance at 340 nm was monitored for 1 h with intervals of 2 min at 37 °C using a Tecan Spark 10 M microplate reader. Enzymatic activities were determined by subtraction of the blank followed by applying the Beer–Lambert law using a molar extinction coefficient for NADH of 6220 L · mol^−1^ · cm^−1^.

### Neu5Ac, Neu5Gc, KDN, and KDO supplementation in HEK293T cells

For sugar supplementation studies, HEK293T cells were seeded in 6-well plates as described above. The next day, cells were transfected with 2 μg of the *NPL* expression vector to overexpress NPL. As a control, WT and NPL KO cell lines were transfected with an empty vector. The feeding was started after one day by replacing the culture medium with fresh medium as the control, or medium supplemented with 1 mM Neu5Ac (Biosynth), Neu5Gc (Biosynth), KDN (Sigma-Aldrich), or KDO (Sigma-Aldrich). Cells were incubated for 24 h in supplemented medium and then snap frozen for metabolite extraction as described below. For the experiment with multiple time points, cells were snap frozen immediately upon supplementation (0 h) or after 2, 4, 6, or 24 h of culture.

### Extraction of polar metabolites from HEK293T cells

Polar metabolites were extracted from HEK293T cells cultured in 6-well plates as previously described by van Scherpenzeel et al. (2022). In brief, cells were carefully washed twice with 75 mM ammonium carbonate (pH 7.2–7.4). Next, the plates were snap frozen in liquid nitrogen and stored at −80 °C until metabolite extraction. As internal standard, 15.3 μM of *N*-acetyl-d-[1,2,3-^13^C_3_]neuraminic acid (^13^C-Neu5Ac; Sigma-Aldrich) was added to each 6-well. For extraction, two rounds were performed of adding 700 μl ice-cold methanol/acetonitrile/water (40:40:20, v/v/v) solution to each well and incubating for 2 min before collecting the extraction solution. In the second round, the incubation period was 3 min. The pooled extracts were centrifuged (19,000 *g*, 3 min, 4 °C) and the supernatant was transferred to a new tube. The supernatants were dried overnight at room temperature in a vacuum concentrator (SC210A Savant™ SpeedVac™, Thermo Scientific). The dried metabolites were stored at −80 °C until use.

### Extraction of polar metabolites from red blood cells

Isolated red blood cells from NPL patient and healthy controls (control 1 and 2) were available at the Translational Metabolic Laboratory (Radboudumc) for previous diagnostic studies (Wen et al. 2018), and used in accordance with Helsinki’s Declaration under Radboudumc local ethical approval number 2019–5591. Additional blood samples from healthy individuals (control 3–5) were received fully de-identified from the blood bank (Sanquin, the Netherlands, Nijmegen). Whole blood was collected in lithium heparin tubes (BD Vacutainer) and used for isolation of red blood cells. Mouse red blood cells were obtained from WT and *Npl^R63C^* knock-in mice generated in a previous study (Da Silva et al. 2023). Approval of animal experiments was previously obtained from the Animal Care and Use Committee of Centre Hospitalier Universitaire (CHU) Sainte-Justine Research Centre (approval numbers: 2020–2668 and 2021–3213). For the isolation of red blood cells, a dextran solution (30 mg/L dextran from *Leuconostoc* spp., 450–600 kDa, in 0.15 M sodium chloride) was added to the blood at a 1:5 ratio and gently mixed. The tube was left upright for 45 min to let the red blood cells sediment. The red blood cells were collected from the lower layer and stored at −80 °C until metabolite extraction. Polar metabolites were extracted by adding ice-cold methanol/acetonitrile/water (75:20:5, v/v/v) solution to red blood cells in a 4:1 ratio (extraction solvent:red blood cell suspension). Samples were incubated for 5 min on dry ice while rotating (300 rpm). Cells were spun down for 15 min at 19,000 *g* and 4 °C. The supernatant was collected and dried overnight at room temperature in a vacuum concentrator (SC210A Savant™ SpeedVac™). The dried metabolites were stored at −80 °C until use.

The remaining pellet from metabolite extraction was used to determine haemoglobin levels. First, pellets were reconstituted in 100 μl MQ water containing 0.5% formic acid and 5% acetonitrile. Subsequently, the samples were heated for 10 min at 95 °C and sonicated for 10 min. Finally, the samples were centrifuged for 20 min at 19,000 *g* and the supernatant was used to determine haemoglobin levels by measuring absorbance of the Soret peak at 405 nm using a NanoDrop 1000 spectrophotometer (Thermo Scientific).

### Polar metabolite analysis by LC–MS/MS and data analysis

Acquisition of sugar metabolites was performed using an Agilent 1290 Infinity ultrahigh-performance liquid chromatography system coupled to an Agilent 6490 quadrupole mass spectrometry system. Metabolite separation was achieved using ion-pair reversed-phase chromatography and a Waters Acquity HSS T3 column (150 × 2.1 mm i.d., 1.8 μm particle size, 100 Å pore size) connected to a Waters Acquity HSS T3 VanGuard pre-column (5 × 2.1 mm i.d., 1.8 μm particle size, 100 Å pore size). Before analysis, dried metabolite extracts were reconstituted in 100 μl MQ water and centrifuged (19,000 *g*, 3 min, 4 °C). The supernatant was loaded into V-bottom 96-well plates. Nucleotide sugars in 8 μl metabolite extract were measured with the triethylamine (TEA)-based method as described previously (van Scherpenzeel et al. 2022) and with transitions added for CMP-KDN and CMP-KDO (Table S2). Additional polar metabolites were measured in 5 μl metabolite extract with the tributylamine (TBA)-based method as adapted from van Scherpenzeel et al. (2022) and with transitions added for KDN and KDO (Table S3). Deviations from the previously described TBA-based method are outlined. Mobile phase A consisted of 10 mM TBA, 12 mM acetic acid, 2 mM acetylacetone, and 3% (v/v) methanol in MQ water. Mobile phase B consisted of 10 mM TBA, 12 mM acetic acid, 2 mM acetylacetone, 3% (v/v) methanol, and 80% (v/v) acetonitrile in MQ water. The column temperature was maintained at 40 °C and a flow rate of 0.5 mL/min was used. After an initial time at 0% B of 11 minutes, a gradient of 3 minutes was run to 15% B. This was followed by a gradient of 5 minutes to 40% B and a gradient of 1 minute to 100% B. The column was flushed at 100% B for 1 minute. After a return to 0% B in 0.5 minute, the column was equilibrated for 3.5 minutes. The total analysis time was 25 minutes. Commercial standards were included in three different mixes during the run. The data obtained was processed using Skyline (v21.2) (Adams et al. 2020). The sialic acid concentration in samples was determined using a ^13^C-Neu5Ac calibration curve (Supplementary material and Fig. S11). Quantification, data normalization, and statistical analysis are described in detail in the Supplementary material.

## Supplementary Material

Supplementary_materials_cwag038

Datasets_S1-S4_cwag038

## Data Availability

The data underlying this article are available in the article and in its online supplementary material.
