# Supplementary material for: Cellular homeostasis of N-acetylneuraminic acid and non-canonical sialic acids is mediated by human N-acetylneuraminate lyase
Source: Glycobiology. 2026 May 18;36(7):cwag038. doi: 10.1093/glycob/cwag038 (PMC13220900; doi:10.1093/glycob/cwag038)
Supplement: Supplementary_materials_cwag038 [file supplementary_materials_cwag038.pdf]

Supplementary Material

Cellular homeostasis of *N*-acetylneuraminic acid and non-canonical sialic acids is mediated by human *N*-acetylneuraminate lyase

Sjanie Huang[1,2], Iris Harmsen[2,9], Moritz Rahm[1,2], Takfarinas Kentache[3], Clara D.M. van Karnebeek[4,5], Afitz Da Silva[6], Alexey V. Pshezhetsky[6,7], Emile Van Schaftingen[3], Alejandro Garanto[2,8], Dirk J. Lefeber[1,2,4*]

1. Department of Neurology, Donders Institute for Brain, Cognition and Behaviour, Radboud University Medical Center, Geert Grooteplein 10, 6525 GA, Nijmegen, The Netherlands
2. Department of Human Genetics, Radboud University Medical Center, Geert Grooteplein 10, 6525 GA, Nijmegen, The Netherlands
3. Laboratory of Physiological Chemistry, De Duve Institute, UCLouvain, Avenue Hippocrate 75, 1200 Brussels, Belgium
4. United for Metabolic Diseases, Meibergdreef 9, 1105 AZ, Amsterdam, The Netherlands
5. Emma Center for Personalized Medicine, Departments of Pediatrics and Human Genetics, Amsterdam UMC, Meibergdreef 9, 1105 AZ, Amsterdam, The Netherlands
6. Department of Pediatrics, Centre Hospitalier Universitaire Sainte-Justine Research Center, University of Montréal, 3175 chemin de la Côte-Sainte-Catherine, Montréal QC H3T 1C5, Canada
7. Department of Anatomy and Cell Biology, McGill University, 3640 University Street, Montréal QC H3A 0C7, Canada
8. Department of Pediatrics, Amalia Children's Hospital, Radboud University Medical Center, Geert Grooteplein 10, 6525 GA, The Netherlands
9. Present address: European Research Institute for the Biology of Ageing, University of Groningen, University Medical Center Groningen, Antonius Deusinglaan 1, 9713 AV, Groningen, The Netherlands.

* Corresponding author: Dirk.Lefeber@Radboudumc.nl

This PDF file includes:

Supplementary materials and methods
Figures S1 to S11
Tables S1 to S3
Legends for Datasets S1 to S4
Supplementary references

Other supplementary materials for this manuscript include the following:

Datasets S1 to S4

Supplementary materials and methods

Bacterial strains

The clones encoding Neu5Ac aldolase (*NanA*; EC 2.5.1.56) and Neu5Ac mutarotase (*NanM*; EC 5.1.3.24) were obtained from the ASKA library, which comprises a complete set of ORF clones of *E. coli* K-12 strain (Kitagawa et al. 2005). ORFs were cloned into the pCA24N vector harbouring a chloramphenicol resistance cassette and a His6-tag located at the N-terminal side of the cloning multiple site.

Cloning of human *NPL* in pET22b(+) vector

The human *NPL* gene was cloned into the pET22b(+) vector as previously described (Kentache et al. 2021). In summary, *NPL* was amplified from human liver complementary DNA (cDNA) by polymerase chain reaction (PCR) using Phusion® DNA polymerase (Thermo Scientific) with forward and reverse primers (hNPL_1; Integrated DNA technologies; IDT) as listed in **Table S1**. The restriction enzymes NdeI and NotI (Thermo Scientific) were used to digest the purified PCR product. The digested product was then ligated into predigested pET22b(+) vector and transformed into chemically competent *Escherichia coli* XL1 Blue. Transformed clones were selected and grown to extract plasmid using Wizard® *Plus* SV minipreps kit (Promega). The sequence was confirmed by Sanger sequencing. Subsequently, the recombinant vector was transformed into chemically competent *E. coli* BL21(DE3) strain, which is more suitable for protein expression.

Expression and purification of recombinant enzymes in *E. coli*

NanA, NanM, and NPL were expressed as previously described (Kentache et al. 2021). For NPL expression, *E. coli* BL21(DE3) recombinant strains were cultured in 200 mL Luria-Bertani (LB) medium (10 g/L tryptone, 5 g/L yeast extract, 10 g/L NaCl) supplemented with 200 µg/mL ampicillin (25 µg/mL chloramphenicol for the expression of NanA and NanM). Expression of the enzymes was induced by adding 0.1 mM of isopropyl-1-thio-β-D-galactopyranoside (IPTG) to bacterial cultures with an optical density (OD) of 0.4 to 0.6 measured at 600 nm. This was followed by incubation at 37 °C for 20 h while shaking at 200 rpm. The cells were harvested by centrifugation at 6,000 *g* for 20 min. Pelleted cells were resuspended in lysis buffer (25 mM HEPES pH 7.5, 10 M KCl, 2 µg/mL antipain and leupeptin, 0.5 mM phenylmethylsulfonyl fluoride [PMSF], 1 mM $MgCl_2$, 1 mM dithiothreitol [DTT]) and subjected to two freeze-thaw cycles in liquid nitrogen, treated with DNAse I (0.1 mg/mL in 10 mM $MgSO_4$) for 30 min on ice and centrifuged at 20,000 *g* for 1 h at 4 °C. The supernatants were kept for protein purification.

His6-tagged protein purifications were performed using the AKTA Purifier 900 series (GE Healthcare) by using a Ni^{2+}-resin column (HisTrap-HP 1 mL, GE Healthcare). The mobile phase consisted of equilibration buffer (10 mM imidazole, 0.3 M NaCl, 30 mM HEPES pH 7.5, 2 µg/mL antipain and leupeptin) and elution buffer (500 mM imidazole, 0.3 M NaCl, 30 mM HEPES pH 7.5, 2 µg/mL antipain and leupeptin). Retained proteins were eluted at a flow rate of 1 mL/min, using a one-step linear gradient from 0 to 100% of elution buffer over 30 min. The 1-milliliter fractions

containing the purified proteins were pooled and concentrated to 2.5 mL using VivaSpin 15 centrifugal filter units according to the manufacturer's recommendations (Sartorius Stedim). After that, desalting was performed using PD-10 Sephadex™ G-25 M columns (GE Healthcare). Proteins were collected in elution buffer (30 mM HEPES pH 7.5, 300 mM KCl, 1 mM DTT, 2 µg/mL antipain and leupeptin). Glycerol was added to the purified proteins at 10% and aliquots were stored at -80 °C for further analyses. Purity of the recombinant proteins was estimated by SDS-PAGE and Coomassie Blue staining. SDS-PAGE was performed in commercial NuPage™ 10% BisTris-glycine gels (Invitrogen) with PageRuler™ plus prestained protein ladder, 10 to 180 kDa (Thermo Scientific) as molecular weight standard. Gels were stained with Coomassie Blue R-250. Protein concentration in the purified preparations was estimated by measuring the absorbance at 280 nm (A_{280}) and computing the concentration from the expected extinction coefficient on the basis of the amino acid composition (Protparam tool; https://web.expasy.org/protparam/).

RNA extraction and quantitative real-time PCR (qRT-PCR)

From three different passages, WT and NPL KO HEK293T cells were collected and lysed in TRIzol™ reagent (Invitrogen) to determine *NPL* gene expression levels. The Direct-zol™ RNA Miniprep kit (Zymo Research) was used to isolate RNA following manufacturer's instructions. Next, 1 µg of total RNA from each sample was reverse transcribed into cDNA using the RevertAid first strand cDNA synthesis kit (Thermo Scientific) according to manufacturer's instructions. Synthesized cDNA was diluted in nuclease-free water (Promega) to 100 ng/µl and used as template in qRT-PCR together with GoTaq® qPCR Master Mix (Promega) in a final reaction volume of 20 µl. Samples were analysed in triplicate using the CFX96 Touch Real-Time PCR detection system (Bio-Rad). No-template controls were included as negative control. Relative *NPL* mRNA expression levels were calculated using the $2^{\wedge-\Delta\Delta Ct}$ method, first against *GUSB* (reference gene) and then against the WT *NPL* levels (Livak and Schmittgen 2001). Primer sequences for *GUSB* and *NPL* are listed in **Table S1**.

Western blotting of NPL

HEK293T cells (WT, NPL KO, and NPL OE) were grown in 6-well plates, pelleted, and lysed in 150 µl RIPA buffer (50 mM Tris-HCl pH 7.5, 150 mM NaCl, 1.0% Triton X-100, 0.5% sodium deoxycholate, 0.1% sodium dodecyl sulphate) supplemented with cOmplete™ protease inhibitor cocktail (Roche) for 15 minutes at 4 °C while rotating. Cell lysates were centrifuged at 19,000 *g* for 10 min at 4 °C. The supernatant was collected and protein concentrations were quantified using the Pierce™ BCA protein assay kit (Thermo Scientific) following manufacturer's instructions. Cell lysates containing 2-20 µg protein were diluted in 4x Laemmli sample buffer (Bio-Rad) supplemented with 10% DTT (1 M) and heated for 5 min at 95 °C. For the western blot where NPL OE cell lysate was included as the positive control, only 0.1 µg protein was used. Samples were loaded onto a 4-15% Mini-PROTEAN™ TGX Stain-Free™ protein gel (Bio-Rad) and run for 30 min at 200 V in Tris-glycine running buffer (Bio-Rad) to separate proteins. Proteins were transferred to a 0.2 µm nitrocellulose membrane (Bio-Rad). Blots were blocked in 5% non-fat dry milk (NFDM; Chemcruz) in Tris-buffered saline with 0.1% Tween-20 (TBST) for 1 h at room temperature (RT). Blots were incubated with the primary antibody rabbit anti-NPL (1:5000; PA5-110039; Thermo

Scientific) diluted in 2% NFDM in TBST at 4 °C overnight or with mouse anti-α-tubulin (1:2000; ab7291; abcam) diluted in 1% NFDM in TBST at RT for 1 h. The used horseradish peroxidase (HRP)-conjugated secondary antibodies were goat anti-rabbit IgG-HRP (1:10,000; A00098; GenScript) and goat anti-mouse IgG-HRP (1:10,000; 12-349; Upstate), both diluted in 1% NFDM in TBST and incubated with the blot for 1 h at RT. Protein bands were visualized using the enhanced chemiluminescent (ECL) SuperSignal™ West Femto kit (Thermo Scientific) according to manufacturer's instructions and the ChemiDOC Touch imaging system (Bio-Rad).

Sialic acid quantification and data analysis

Sialic acid concentrations were quantified by LC-MS/MS using an Agilent 1290 infinity II ultrahigh-performance liquid chromatography system coupled to a Sciex 6500+ QTRAP mass spectrometer. Separation was achieved on a Waters Atlantis Premier BEH C18 AX Column (2.1 × 150 mm, 1.7 µm particle size; 50 °C) and a flow rate of 0.5 ml/min. The mobile phase A consisted of MQ, and mobile phase B consisted of 50 mM ammonium acetate, 50 mM acetic acid, and 5% acetonitrile in MQ. The used gradient program was as follows: 0-1.6 min, 5% B; 2.5 min, 30% B; 2.7-4 min, 100% B; 4.1-6 min, 5% B. To construct a calibration curve, standards with a range of concentrations of ^{13}C-Neu5Ac (0.0, 0.005, 0.010, 0.025, 0.050, 0.10, 0.25, 0.50, 1.0, 2.5, 7.5, 15 µM) were included during the LC-MS/MS measurement. The obtained peak intensities of Neu5Ac, Neu5Gc, KDN, and KDO were normalized using the internal standard. A normalization factor was calculated per sample as follows: normalization factor = observed peak area of internal standard in sample / highest observed peak area of internal standard in all measured samples. Next, the normalized peak area was determined by dividing the peak area of the metabolite of interest by the normalization factor. After normalization, the concentration of Neu5Ac, Neu5Gc, and KDN was calculated based on the constructed calibration curve (**Fig. S11**). Haemoglobin levels were used to normalize metabolite data from red blood cells. Additional metabolite data from HEK293T samples was represented as the normalized peak area. For this, the obtained raw peak area from each individual metabolite was divided by the total peak area of that sample. Neu5Ac, Neu5Gc, KDN, and KDO were excluded from the total peak area calculation, since these sugars were also introduced during feeding conditions. GraphPad Prism (v10.4.1) was used for data visualization and statistical analysis. Normality of the data was assessed using the Shapiro-Wilk test. A two-tailed unpaired t-test was used to compare the mean of two groups when data had a normal distribution, otherwise the Kolmogorov-Smirnov test was used.

Synthesis of CMP-KDN and CMP-Neu5Gc

Standards for CMP-KDN and CMP-Neu5Gc were synthesized as previously described (Gilormini et al. 2016). Briefly, KDN or Neu5Gc, and CTP disodium salt (Sigma-Aldrich) were dissolved in 100 mM Tris-HCl buffer (pH = 8.8) containing 50 mM $MgCl_2$ and 0.2 mM DTT. To start the reaction, CMP-sialic acid synthetase (CMAS) from *Neisseria meningitidis* group B (EC 2.7.7.43; 1 U) and inorganic pyrophosphatase from *Saccharomyces cerevisiae* (EC 3.6.1.1; 1 U) were added to the reaction mixture, which was incubated for a minimum of 4 h at 37 °C with shaking (300 rpm). The mixture was spun down (10,000 *g*, 10 min). The supernatant was collected to use as standard and stored at -80 °C. Synthesized products were confirmed by negative mode LC-MS/MS (**Fig. S7**). The

precursors anions of CMP-Neu5Ac, CMP-Neu5Gc, CMP-KDN, and CMP-KDO were tracked at 613.1 *m/z*, 629.1 *m/z*, 572.1 *m/z*, and 542.0 *m/z*, respectively (**Table S2**). The following product ion *m/z* values were monitored to confirm identity of the synthesized molecules: 79.0 *m/z* indicating the metaphosphate anion $[PO_3]^-$ fragment, 97.0 *m/z* indicating the dihydrogen phosphate anion $[PO_4H_2]^-$ fragment, 322.0 *m/z* indicating the CMP anion $[C_9H_{13}N_3O_8P]^-$ fragment. Additionally, a fragment representing Sia-P^--H_2O was recorded for each CMP-sialic acid, with CMP-Neu5Ac at 370.1 m/z $[C_{11}H_{17}NO_{11}P]^-$, CMP-Neu5Gc at 386.0 *m/z* $[C_{11}H_{17}NO_{12}P]^-$, CMP-KDN at 329.0 *m/z* $[C_9H_{14}O_{11}P]^-$, and CMP-KDO at 298.0 *m/z* $[C_8H_{12}O_{10}P]^-$. Synthesis of CMP-KDO was attempted following the same procedure, but was not successful. Failed CMP-KDO synthesis might be explained by the short stability of CMP-KDO (Sugai et al. 1995).

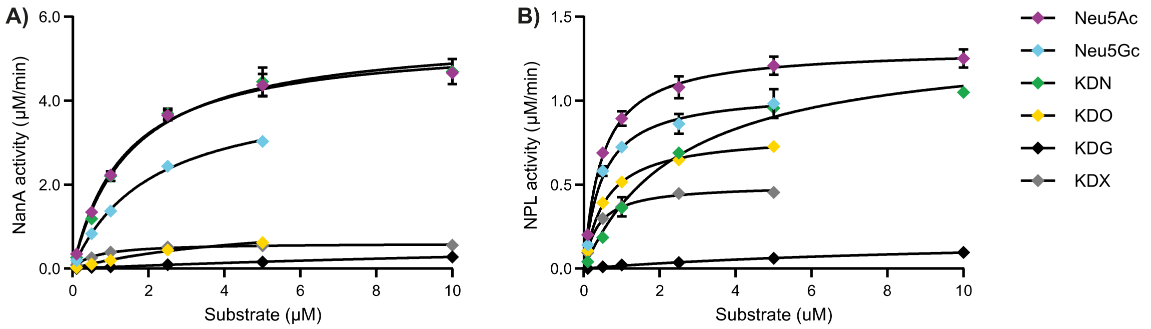

Figure S1. ***E. coli*** **NanA and human NPL activity towards different sialic acids and structurally similar sugars.** Michaelis-Menten plots showing kinetic curves of (**A**) *E. coli* NanA and (**B**) human NPL determined by an NADH-coupled enzyme assay towards different substrates. Data are represented as the mean ± SEM.

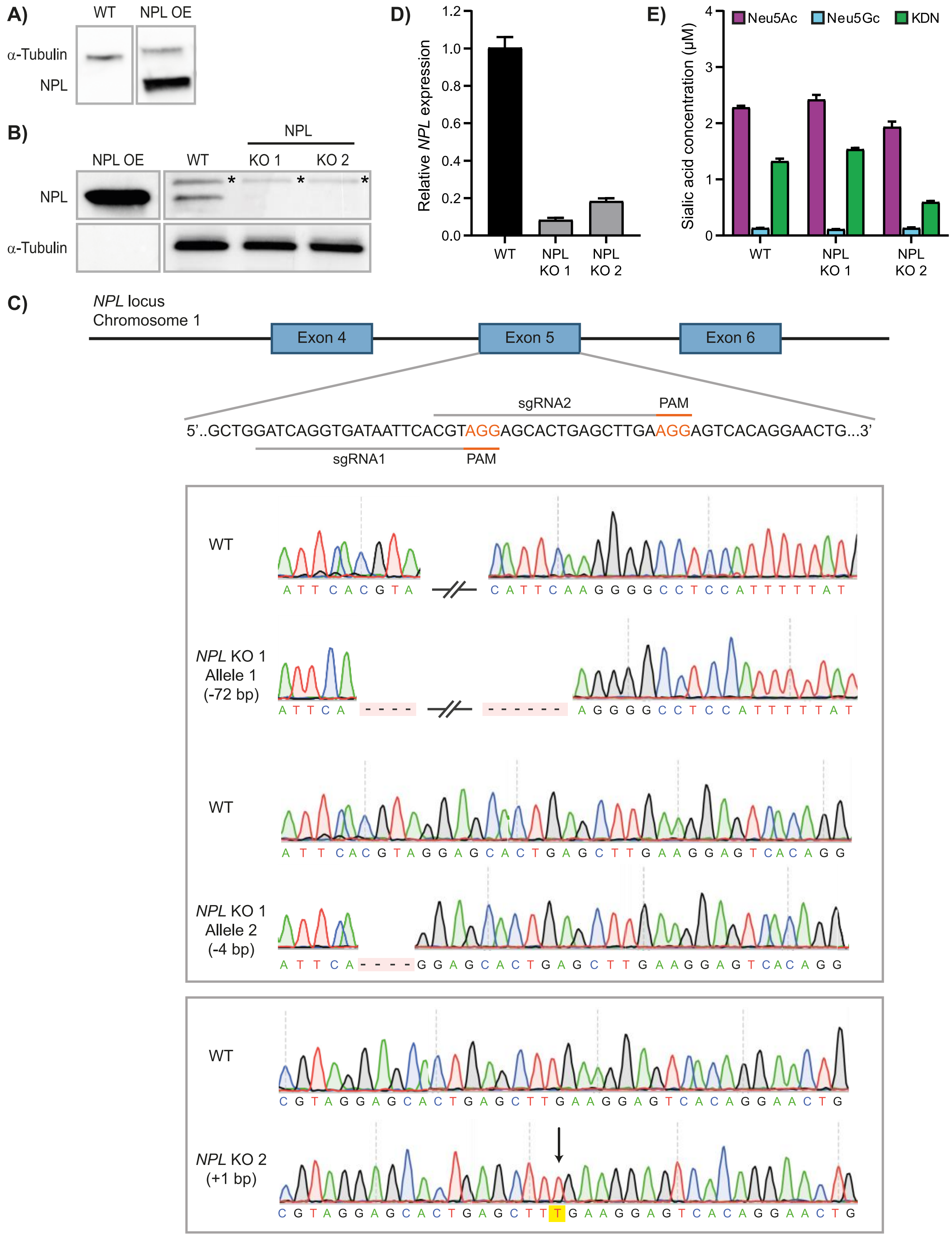

Figure S2. **Generation of HEK293T *NPL* KO and *NPL* OE cell models.** (**A**) Overexpression of *NPL* successfully increased levels of NPL protein as shown by western blot. The NPL protein band was not visible in the WT because imaging conditions were optimized to avoid oversaturation of the OE band, which resulted in the WT band being below the detection threshold. α-Tubulin was used as loading control and 10 µg of protein was loaded per sample. (**B**) NPL protein was undetectable in the NPL KO lines as determined by western blot. Lysate from HEK293T cells overexpressing NPL was used as the positive control, which was loaded with

200 times less protein (0.1 ug) than the other samples (20 µg). The asterisk (*) marks a non-specific band. (**C**) Two *NPL* KO clones in HEK293T were generated using CRISPR/Cas9 with two different sgRNAs targeting exon 5 of *NPL*. Sanger sequencing showed that *NPL* KO 1 carried heterozygous mutations: one allele with a 72 bp deletion and one allele with a 4 bp deletion. *NPL* KO 2 was homozygous for a single T insertion. (**D**) Both *NPL* KO lines showed reduced relative *NPL* mRNA expression compared to the WT. Data are represented as the mean ± SD of three biological replicates. (**E**) Neu5Ac, Neu5Gc, and KDN levels were determined in HEK293T NPL KO cells by LC-MS/MS using ^{13}C-Neu5Ac as internal standard. Data are represented as the mean of three biological replicates ± SD.

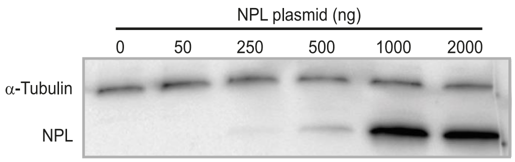

Figure S3. **Dose-dependent increase in NPL protein levels induced by transfection of HEK293T cells with NPL plasmid.** NPL protein levels were gradually increased with increasing doses of NPL plasmid (0-2000 ng) as shown by western blot. The NPL protein bands for cells transfected with 0 ng and 50 ng NPL plasmid were below the detection threshold as imaging conditions were optimized to avoid oversaturation of the 1000 ng and 2000 ng bands. α-Tubulin was used as loading control and 2 μg of protein was loaded per sample.

A)

Potential off-target	Chr	Strand	Start	End	sgRNA	Potential off-target sequence	Number of mismatches
1	7	-	118679032	118679054	sgRNA1	GATCAtcTGATAAaTCACGTTGG	3
2	4	-	89870460	89870482	sgRNA1	GATCAGGTGATAATgCAatTAGG	3
3	2	-	97563680	97563702	sgRNA2	CGTtGGAGaACTGAaCTTGAAGG	3
4	21	-	33380758	33380780	sgRNA2	gGgAGGAGaACTGAGCTTGAAGG	3
5	5	-	178931527	178931549	sgRNA2	CtgAGGAGCACTGAGCTTGgAGG	3
6	6	-	86651036	86651058	sgRNA2	CcTAGGAGCcCTGAGCTTcATGG	3

B)

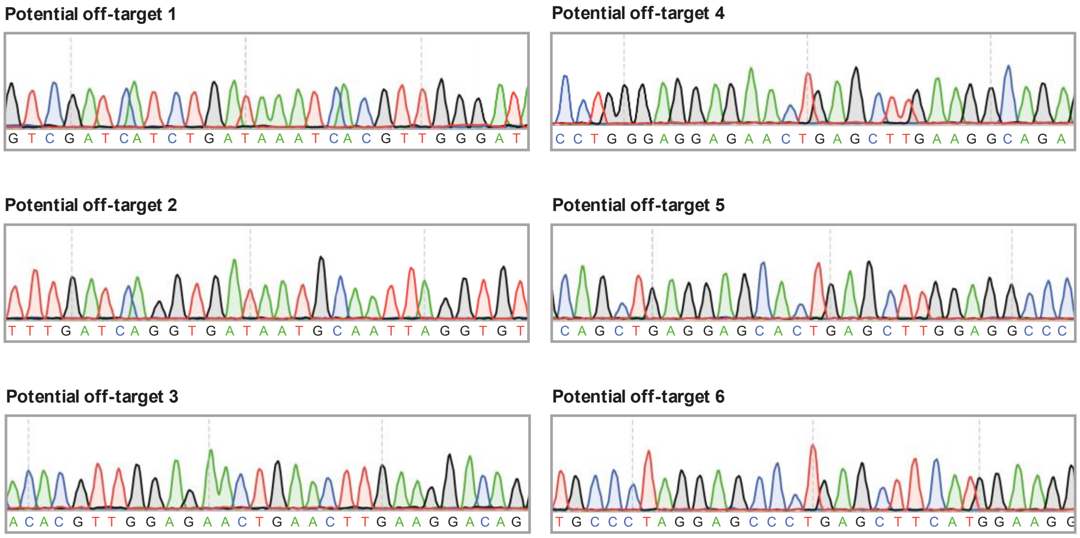

Figure S4. Off-target analysis of generation of the *NPL* KO clones. (**A**) Table showing the predicted potential off-targets for sgRNA1 and sgRNA2, including their sequence, chromosome location, and the number of mismatches. Mismatches are indicated by lowercase letters and the PAM is underlined in the off-target sequence. (**B**) Sanger sequencing results showed that no editing at the potential off-target sites has occurred.

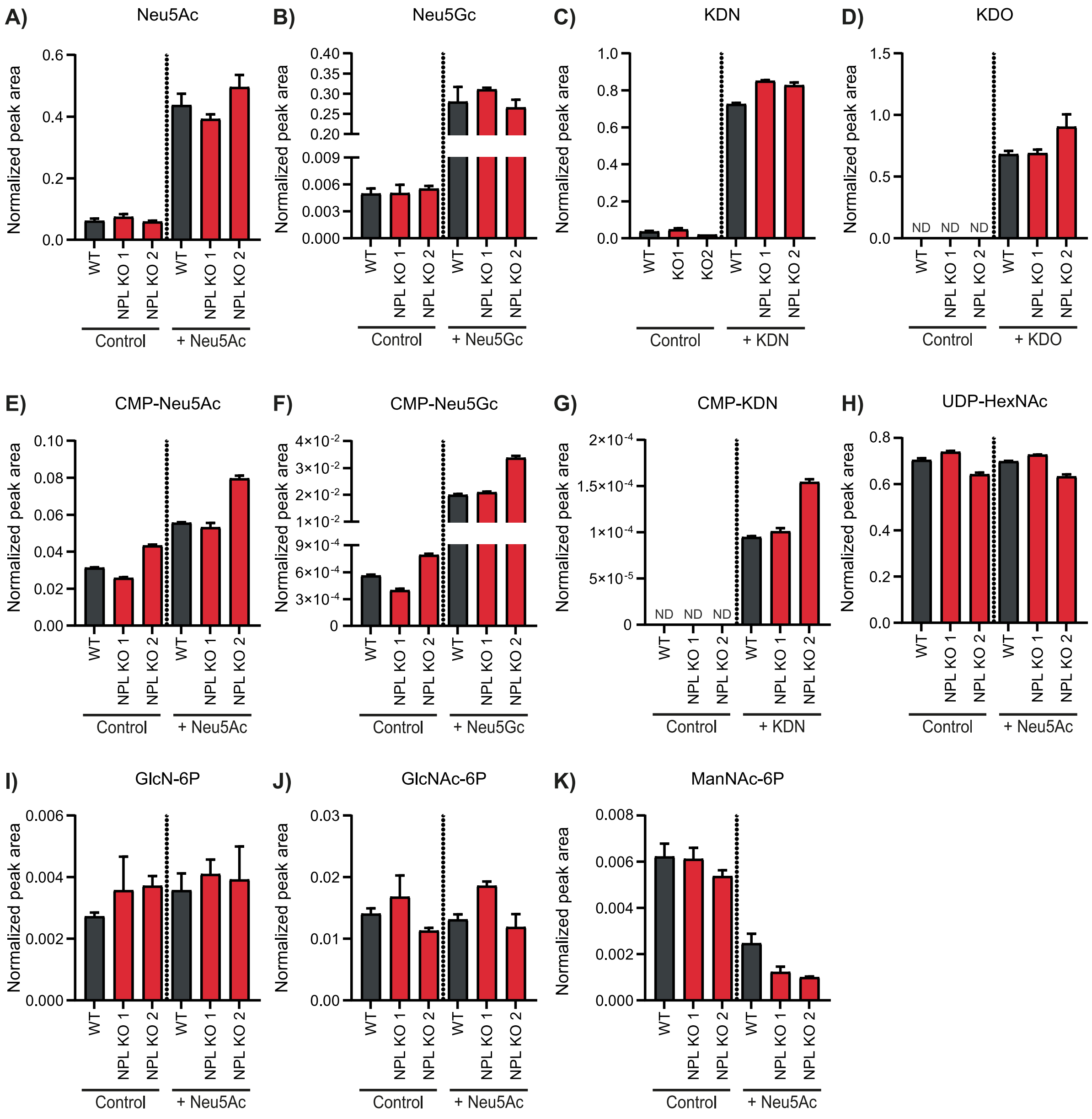

Figure S5. Sialic acid pathway metabolites assessed in WT and NPL KO HEK293T cells. Normalized peak areas of (**A**) Neu5Ac, (**B**) Neu5Gc, (**C**) KDN, (**D**) KDO, (**E**) CMP-Neu5Ac, (**F**) CMP-Neu5Gc, (**G**) CMP-KDN, (**H**) UDP-HexNAc, (**I**) GlcN-6P, (**J**) GlcNAc-6P, and (**K**) ManNAc-6P in HEK293T NPL KO cells cultured for 24 h in medium without supplementation (control) or with supplementation of 1 mM Neu5Ac, Neu5Gc, KDN, or KDO respectively. Neu5Ac-9P was not detected in WT and NPL KO cells in control and supplemented conditions. Data are represented as the mean of three biological replicates ± SD. ND indicates that the metabolite level in the sample was below the limit of detection.

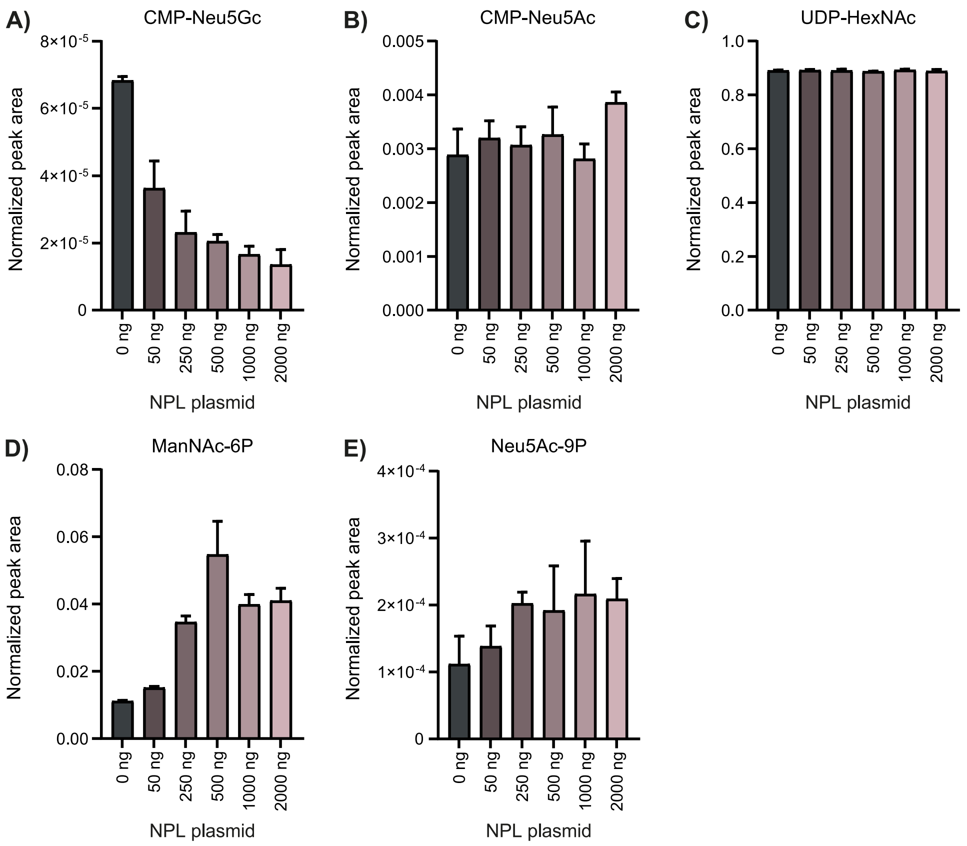

Figure S6. Sialic acid pathway metabolite levels upon overexpression of different doses NPL plasmid in HEK293T cells. Normalized peak areas of (**A**) CMP-Neu5Gc, (**B**) CMP-Neu5Ac, (**C**) UDP-HexNAc, (**D**) ManNAc-6P, and (**E**) Neu5Ac-9P were determined in HEK293T cells transfected with 0-2000 ng NPL plasmid. Nucleotide sugars were quantified as previously described (Rahm et al. 2024) and phosphate sugars were quantified using the described TBA-based method, but adapted to analysis on a Waters M-class UPLC system connected to a Waters Xevo TQS system with a Waters Acquity UPLC HSS T3 column (150 × 1.0 mm i.d., 1.8 µm particle size, 100 Å pore size). Data are represented as the mean of three biological replicates ± SD.

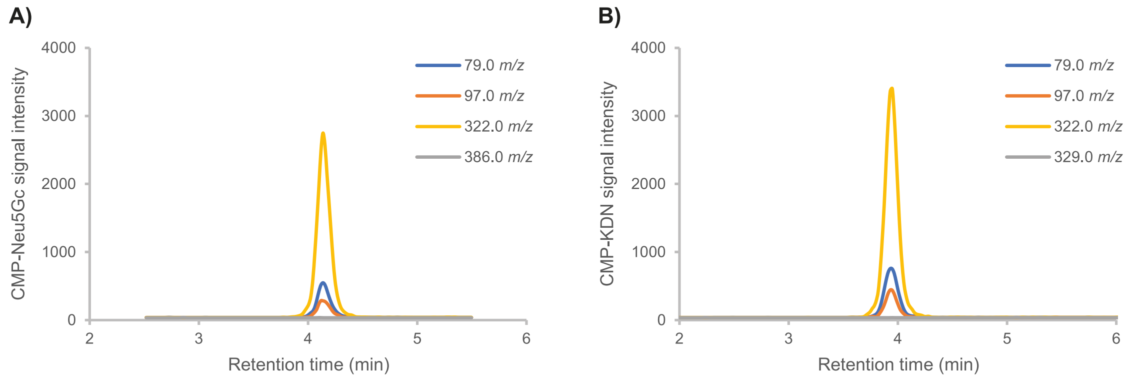

Figure S7. Identification of CMP-Neu5Gc and CMP-KDN. Multiple reaction monitoring (MRM) chromatograms for synthesized (**A**) CMP-Neu5Gc and (**B**) synthesized CMP-KDN detected by LC/MS-MS.

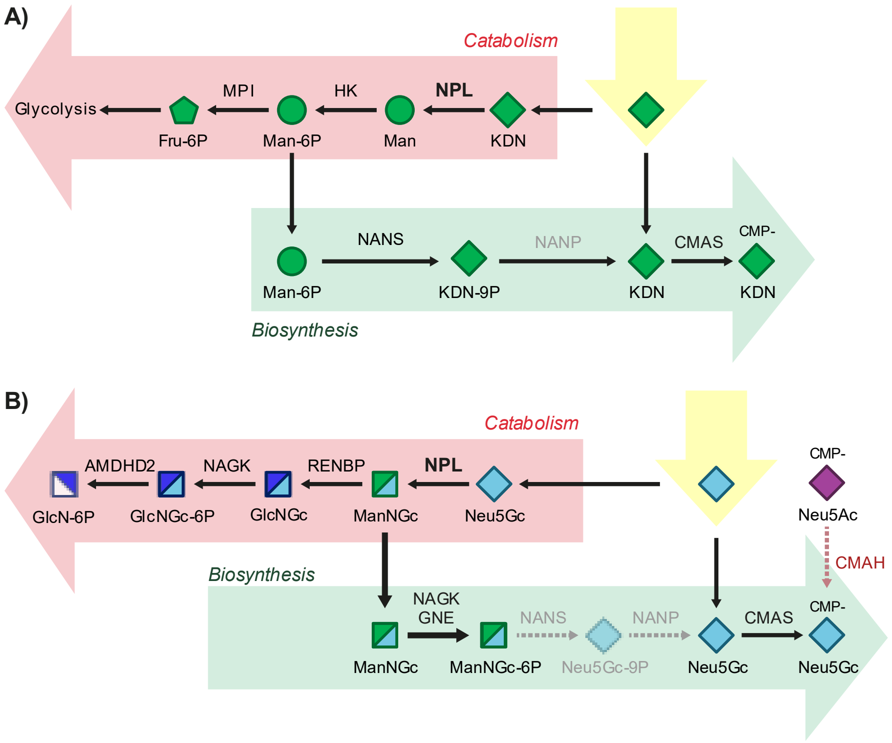

Figure S8. Presumed metabolic pathways of KDN and Neu5Gc catabolism and biosynthesis. A) NPL catabolizes KDN to mannose (Man) and pyruvate. Mannose is phosphorylated by hexokinase (HK) to mannose 6-phosphate (Man-6P), which could be converted to fructose 6-phosphate (Fru-6P) by mannose-6 phosphate isomerase (MPI) to enter glycolysis. Alternatively, Man-6P could be converted to KDN-9P by *N*-acetylneuraminic acid synthase (NANS) for KDN synthesis. Dephosphorylation of KDN-9P is likely catalysed by *N*-acetylneuraminic acid phosphatase (NANP), yielding KDN, which could be activated by CMP-sialic acid synthetase (CMAS) to obtain CMP-KDN. **B**) NPL catabolizes Neu5Gc to ManNGc and pyruvate. ManNGc could be converted to GlcNGc by *N*-acetyl-D-glucosamine 2-epimerase (RENBP), followed by phosphorylation to GlcNGc-6P by *N*-acetyl-D-glucosamine kinase (NAGK). Alternatively, ManNGc could be phosphorylated to ManNGc-6P by NAGK or by the kinase domain of GNE (UDP-*N*-acetylglucosamine 2-epimerase/*N*-acetylmannosamine kinase). It remains unclear whether ManNGc-6P could be further metabolized by NANS and NANP to obtain Neu5Gc via Neu5Gc-9P. However, Neu5Gc-9P was not detected in HEK293T cells. When Neu5Gc is not directly catabolized by NPL, it can be activated by CMAS to obtain CMP-Neu5Gc. Humans are not able to *de novo* synthesize CMP-Neu5Gc from CMP-Neu5Ac due to loss of CMP-*N*-acetylneuraminic acid hydroxylase (CMAH) function.

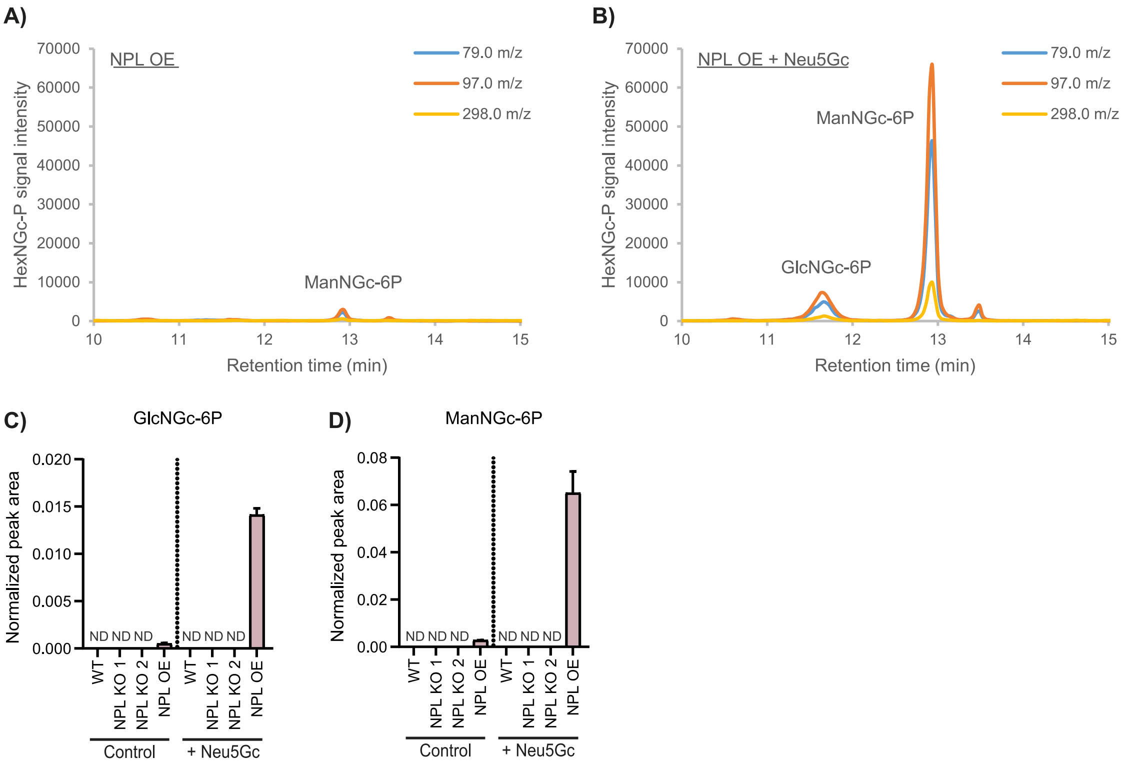

Figure S9. Increased *N*-glycolylhexosamine 6-phosphate levels were detected in HEK293T cells upon NPL overexpression. (**A-B**) Multiple reaction monitoring (MRM) chromatograms for *N*-glycolylmannosamine 6-phosphate (ManNGc-6P) and *N*-glycolylglucosamine 6-phosphate (GlcNGc-6P) detected by LC/MS-MS. The peak identities were based on their similarity to HexNAc-P elution profiles and fragmentation, since internal standards are not available. (**A**) Detection of a ManNGc-6P peak in NPL OE cells without supplementation as a result of NPL-mediated cleavage of endogenous Neu5Gc in HEK293T cells. (**B**) Detection of GlcNGc-6P (11.7 min) and an elevated ManNGc-6P peak (12.9 min) in NPL OE cells supplemented with 1 mM Neu5Gc, as a result of NPL-mediated cleavage of supplemented Neu5Gc. Normalized peak areas of (**C**) GlcNGc-6P and (**D**) ManNGc-6P in the different HEK293T cell models cultured for 24 h in medium without supplementation (control) or with supplementation of 1 mM Neu5Gc. Data are represented as the mean of three biological replicates ± SD. ND indicates that the metabolite level in the sample was below the limit of detection.

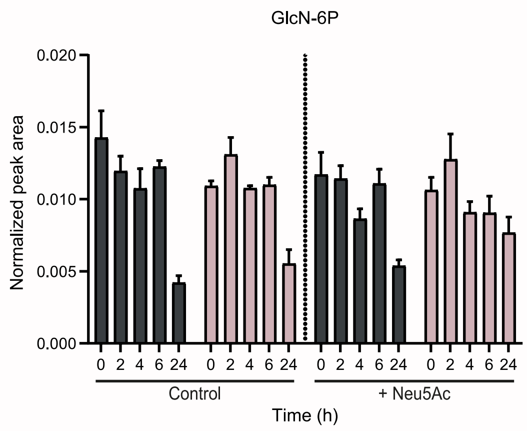

Figure S10. GlcN-6P levels over time upon NPL overexpression in control and Neu5Ac supplemented HEK293T cells. Normalized peak area of GlcN-6P in WT and NPL OE cells after 0, 2, 4, 6, and 24 h in culture medium without supplementation (control) or with supplementation of 1 mM Neu5Ac. Data are represented as the mean of three biological replicates ± SD.

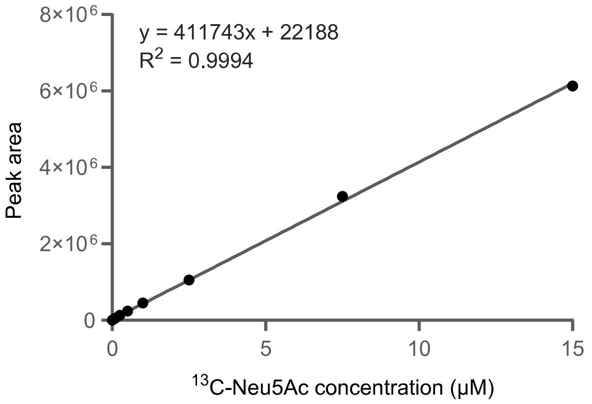

Figure S11. ^{13}C-Neu5Ac calibration curve used to quantify sialic acid concentration. A calibration curve was constructed by measuring the response of ^{13}C-Neu5Ac at a concentration range of 0-15 μM using LC-MS/MS. The response was plotted as peak area versus the known concentration. Coefficient of determination showed very good linearity (R^2 = 0.9994).

Table S1. Overview of used oligonucleotide sequences.

Use	Name	Sequence (5' – 3')
Cloning	hNPL_1	GAGACATATGATGGCCTTCCCAAAGAAGAAA (fw; with NdeI restriction site underlined)
		AAATGCGGCCGCGCTACCAGCTTCCAAGTTTC (rv; with NotI restriction site underlined)
	hNPL_2	gggacaagtttgtacaaaaaagcaggcttcaccatgGCCTTCCCAAAGAAGAAACTTC (fw)
		ggggaccactttgtacaagaaagctgggtctaGCTACCAGCTTCCAAGTT (rv)
CRISPR/Cas9	sgRNA1_NPL	caccgATCAGGTGATAATTCACGT (fw)
		aaacACGTGAATTATCACCTGATc (rv)
	sgRNA2_NPL	caccgCGTAGGAGCACTGAGCTTGA (fw)
		aaacTCAAGCTCAGTGCTCCTACGc (rv)
Sequencing	NPL_exon5_seq	AAACCAGGATAGTGCAGCCC (fw)
		TAAGCCCCTGAGATCCCACA (rv)
Potential off-target check	POT_1	GCCACTTTTCAAGAGCACCTG (fw)
		CGAAAGCCATGGGGTTATTGG (rv)
	POT_2	CTGGCAAGGAACTACATAAGGGA (fw)
		CTCACCTTTGTTTCAATGATGACG (rv)
	POT_3	AATTTGCAGGGCACTGCTGG (fw)
		GTCAAAACCCTCACCGTAGCC (rv)
	POT_4	GCAGGTGATGCAATTGAGACCA (fw)
		CCATCACCATGTCCACCCTTTC (rv)
	POT_5	CCAAGAATGCAAAGCTTGGGC (fw)
		CTCACACAGCAAGCAGAGCG (rv)
	POT_6	GATGGGCTGATCTGCAGAGC (fw)
		CCTTAGTGAGTGGCTTGGCC (rv)
qRT-PCR	NPL	TGTGAAAGAACAGGGAGTGAAGAAC (fw)
		CCCTTTTGTCACCCACTCCTC (rv)
	GUSB	CTGTACACGACACCCACCAC (fw)
		TACAGATAGGCAGGGCGTTC (rv)

Table S2. Multiple reaction monitoring (MRM) transitions and collision energies of nucleotide sugars from the TEA-based method measured by LC-MS/MS. Transitions were based on a previously developed method (van Scherpenzeel et al. 2022) and new transitions were added for CMP-KDN and CMP-KDO.

Compound name	Q1 mass (precursor ion) [amu]	Q3 mass (product ion) [amu]	Retention time [min]	Dynamic MRM time window [min]	Collision energy [eV]
ADP-ribose	558.1	426.0	18.5	18.8 ± 3.0	26.0
	558.1	408.0	18.5	18.8 ± 3.0	27.0
	558.1	346.1	18.5	18.8 ± 3.0	23.0
	558.1	273.0	18.5	18.8 ± 3.0	44.0
	558.1	158.9	18.5	18.8 ± 3.0	44.0
	558.1	97.0	18.5	18.8 ± 3.0	51.0
	558.1	79.0	18.5	18.8 ± 3.0	61.0
CDP-ribitol	536.1	402.0	4.5	4.2 ± 1.5	24.0
	536.1	384.0	4.5	4.2 ± 1.5	26.0
	536.1	322.0	4.5	4.2 ± 1.5	22.0
	536.1	273.0	4.5	4.2 ± 1.5	35.0
	536.1	158.9	4.5	4.2 ± 1.5	45.0
	536.1	97.0	4.5	4.2 ± 1.5	51.0
	536.1	79.0	4.5	4.2 ± 1.5	60.0
CMP-KDN	572.1	329.0	4.0	4.0 ± 3.0	31.0
	572.1	322.0	4.0	4.0 ± 3.0	22.0
	572.1	97.0	4.0	4.0 ± 3.0	51.0
	572.1	79.0	4.0	4.0 ± 3.0	60.0
CMP-KDO	542.0	322.0	N.A.	5.0 ± 3.5	22.0
	542.0	298.0	N.A.	5.0 ± 3.5	31.0
	542.0	97.0	N.A.	5.0 ± 3.5	51.0
	542.0	79.0	N.A.	5.0 ± 3.5	60.0
CMP-Neu5Ac	613.1	370.1	5.1	6.1 ± 1.5	31.0
	613.1	322.0	5.1	6.1 ± 1.5	22.0
	613.1	97.0	5.1	6.1 ± 1.5	51.0
	613.1	79.0	5.1	6.1 ± 1.5	60.0
CMP-Neu5Gc	629.1	386.0	4.2	4.0 ± 1.5	31.0
	629.1	322.0	4.2	4.0 ± 1.5	17.0
	629.1	97.0	4.2	4.0 ± 1.5	53.0
	629.1	79.0	4.2	4.0 ± 1.5	65.0
GDP-fucose	588.1	442.0	16.3	16.8 ± 1.5	24.0
	588.1	424.0	16.3	16.8 ± 1.5	26.0
	588.1	362.1	16.3	16.8 ± 1.5	22.0
	588.1	273.0	16.3	17.8 ± 2.6	36.5
	588.1	225.0	16.3	16.8 ± 1.5	31.0

	588.1	158.9	16.3	17.8 ± 2.6	44.5
	588.1	97.0	16.3	17.8 ± 2.6	51.0
	588.1	79.0	16.3	17.8 ± 2.6	60.0
GDP-mannose	604.1	442.0	11.3	13.0 ± 2.7	24.7
	604.1	424.0	11.3	13.0 ± 2.7	28.3
	604.1	362.1	11.3	13.0 ± 2.7	24.0
	604.1	273.0	11.3	13.0 ± 2.7	39.3
	604.1	241.0	11.3	13.0 ± 2.7	33.7
	604.1	158.9	11.3	13.0 ± 2.7	46.0
	604.1	97.0	11.3	13.0 ± 2.7	51.0
	604.1	79.0	11.3	13.0 ± 2.7	63.3
UDP-arabinose	535.0	403.0	6.1	7.5 ± 2.5	23.0
	535.0	385.0	6.1	7.5 ± 2.5	25.5
	535.0	323.0	6.1	7.5 ± 2.5	21.0
	535.0	273.0	6.1	7.5 ± 2.5	34.5
	535.0	211.0	6.1	7.5 ± 2.5	32.0
	535.0	158.9	6.1	7.5 ± 2.5	44.0
	535.0	97.0	6.1	7.5 ± 2.5	51.0
	535.0	79.0	6.1	7.5 ± 2.5	65.0
UDP-galactose	565.0	403.0	6.3	6.8 ± 2.2	24.0
	565.0	385.0	6.3	6.8 ± 2.2	26.0
	565.0	323.0	6.3	6.8 ± 2.2	22.0
	565.0	273.0	6.3	6.8 ± 2.2	36.0
	565.0	241.0	6.3	6.8 ± 2.2	30.7
	565.0	158.9	6.3	6.8 ± 2.2	45.3
	565.0	97.0	6.3	6.8 ± 2.2	51.0
	565.0	79.0	6.3	6.8 ± 2.2	61.3
UDP-glucose	565.0	403.0	7.0	6.8 ± 2.2	24.0
	565.0	385.0	7.0	6.8 ± 2.2	26.0
	565.0	323.0	7.0	6.8 ± 2.2	22.0
	565.0	273.0	7.0	6.8 ± 2.2	36.0
	565.0	241.0	7.0	6.8 ± 2.2	30.7
	565.0	158.9	7.0	6.8 ± 2.2	45.3
	565.0	97.0	7.0	6.8 ± 2.2	51.0
	565.0	79.0	7.0	6.8 ± 2.2	61.3
UDP-glucuronic acid	579.0	403.0	14.2	15.0 ± 1.5	24.0
	579.0	385.0	14.2	15.0 ± 1.5	28.0
	579.0	323.0	14.2	15.0 ± 1.5	21.0
	579.0	273.0	14.2	15.0 ± 1.5	41.0
	579.0	255.0	14.2	15.0 ± 1.5	23.0
	579.0	158.9	14.2	15.0 ± 1.5	44.0

	579.0	97.0	14.2	15.0 ± 1.5	51.0
	579.0	79.0	14.2	15.0 ± 1.5	80.0
UDP-HexNAc	606.1	403.0	8.8	9.5 ± 1.5	25.3
	606.1	385.0	8.8	9.5 ± 1.5	27.0
	606.1	323.0	8.8	9.5 ± 1.5	27.7
	606.1	282.0	8.8	9.5 ± 1.5	31.7
	606.1	273.0	8.8	9.5 ± 1.5	36.3
	606.1	158.9	8.8	9.5 ± 1.5	50.3
	606.1	97.0	8.8	9.5 ± 1.5	51.0
	606.1	79.0	8.8	9.5 ± 1.5	72.7
UDP-mannose	565.0	403.0	5.7	6.8 ± 2.2	24.0
	565.0	385.0	5.7	6.8 ± 2.2	26.0
	565.0	323.0	5.7	6.8 ± 2.2	22.0
	565.0	273.0	5.7	6.8 ± 2.2	36.0
	565.0	241.0	5.7	6.8 ± 2.2	30.7
	565.0	158.9	5.7	6.8 ± 2.2	45.3
	565.0	97.0	5.7	6.8 ± 2.2	51.0
	565.0	79.0	5.7	6.8 ± 2.2	61.3
UDP-xylose	535.0	403.0	8.0	7.5 ± 2.5	23.0
	535.0	385.0	8.0	7.5 ± 2.5	25.5
	535.0	323.0	8.0	7.5 ± 2.5	21.0
	535.0	273.0	8.0	7.5 ± 2.5	34.5
	535.0	211.0	8.0	7.5 ± 2.5	32.0
	535.0	158.9	8.0	7.5 ± 2.5	44.0
	535.0	97.0	8.0	7.5 ± 2.5	51.0
	535.0	79.0	8.0	7.5 ± 2.5	65.0

Table S3. Multiple reaction monitoring (MRM) transitions and collision energies of polar metabolites from the TBA-based method measured by LC-MS/MS. Transitions were adapted from a previously developed method (van Scherpenzeel et al. 2022).

Compound name	Q1 mass (precursor ion) [amu]	Q3 mass (product ion) [amu]	Retention time [min]	Dynamic MRM time window [min]	Collision energy [eV]
^{13}C-Neu5Ac	311.0	173.0	4.7	4.6 ± 1.5	20.0
	311.0	119.0	4.7	4.6 ± 1.5	17.0
	311.0	101.0	4.7	4.6 ± 1.5	33.0
	311.0	90.0	4.7	4.6 ± 1.5	17.0
Fructose-1P	259.0	169.0	13.7	11.5 ± 3.8	5.8
	259.0	97.0	13.7	11.5 ± 3.8	13.3
	259.0	79.0	13.7	11.5 ± 3.8	50.5
Fructose-6P	259.0	169.0	11.2	11.5 ± 3.8	5.8
	259.0	139.0	11.2	10.7 ± 3.0	10.8
	259.0	97.0	11.2	11.5 ± 3.8	13.3
	259.0	79.0	11.2	11.5 ± 3.8	50.5
Galactonic acid	195.1	129.0	3.9	3.3 ± 1.3	11.0
	195.1	75.0	3.9	3.3 ± 1.3	18.0
Galactose-1P	259.0	241.0	10.9	11.6 ± 2.1	9.0
	259.0	139.0	10.9	10.7 ± 3.0	10.8
	259.0	97.0	10.9	11.5 ± 3.8	13.3
	259.0	79.0	10.9	11.5 ± 3.8	50.5
GalNAc-1P	300.0	97.0	12.3	12.9 ± 1.7	15.5
	300.0	79.0	12.3	12.9 ± 1.7	48.0
GlcNAc-1P	300.0	97.0	12.9	12.9 ± 1.7	15.5
	300.0	79.0	12.9	12.9 ± 1.7	48.0
GlcNAc-6P	300.0	282.0	13.1	13.3 ± 1.2	6.0
	300.0	97.0	13.1	12.9 ± 1.7	15.5
	300.0	79.0	13.1	12.9 ± 1.7	48.0
GlcNGc-6P	316.0	298.0	11.8	13.1 ± 1.7	6.0
	316.0	97.0	11.8	13.1 ± 1.7	17.0
	316.0	79.0	11.8	13.1 ± 1.7	60.0
Gluconic acid	195.1	129.0	4.1	3.3 ± 1.3	11.0
	195.1	75.0	4.1	3.3 ± 1.3	18.0
Glucosamine-6P	258.0	240.0	1.2	0.9 ± 1.5	9.0
	258.0	97.0	1.2	0.9 ± 1.5	15.0
	258.0	79.0	1.2	0.9 ± 1.5	45.0
Glucose-1P	259.0	241.0	12.1	11.6 ± 2.1	9.0

	259.0	139.0	12.1	10.7 ± 3.0	10.8
	259.0	97.0	12.1	11.5 ± 3.8	13.3
	259.0	79.0	12.1	11.5 ± 3.8	50.5
Glucose-6P	259.0	199.0	9.5	9.7 ± 2	7.0
	259.0	169.0	9.5	11.5 ± 3.8	5.8
	259.0	139.0	9.5	10.7 ± 3.0	10.8
	259.0	97.0	9.5	11.5 ± 3.8	13.3
	259.0	79.0	9.5	11.5 ± 3.8	50.5
KDN	267.1	129.0	3.9	3.8 ± 1.5	20.0
	267.1	87.0	3.9	3.8 ± 1.5	28.0
KDO	237.1	129.0	4.2	4.2 ± 3.0	20.0
	237.1	87.0	4.2	4.2 ± 3.0	28.0
ManNAc-6P	300.0	282.0	13.5	13.3 ± 1.2	6.0
	300.0	97.0	13.5	12.9 ± 1.7	15.5
	300.0	79.0	13.5	12.9 ± 1.7	48.0
ManNGc-6P	316.0	298.0	13.0	13.1 ± 1.7	6.0
	316.0	97.0	13.0	13.1 ± 1.7	17.0
	316.0	79.0	13.0	13.1 ± 1.7	60.0
Mannose-1P	259.0	97.0	11.7	11.5 ± 3.8	13.3
	259.0	79.0	11.7	11.5 ± 3.8	50.5
Mannose-6P	259.0	199.0	10.1	9.7 ± 2	7.0
	259.0	169.0	10.1	11.5 ± 3.8	5.8
	259.0	139.0	10.1	10.7 ± 3.0	10.8
	259.0	97.0	10.1	11.5 ± 3.8	13.3
Neu5Ac	308.1	170.0	4.7	4.6 ± 1.5	20.0
	308.1	119.0	4.7	4.6 ± 1.5	17.0
	308.1	98.0	4.7	4.6 ± 1.5	33.0
	308.1	87.0	4.7	4.6 ± 1.5	17.0
Neu5Ac-9P	388.1	300.0	19.0	19.3 ± 2.0	12.0
	388.1	97.0	19.0	19.3 ± 2.0	30.0
	388.1	79.0	19.0	19.3 ± 2.0	50.0
Neu5Gc	324.1	186.0	4.2	4.1 ± 1.5	13.0
	324.1	114.0	4.2	4.1 ± 1.5	20.0
	324.1	87.0	4.2	4.1 ± 1.5	21.0
Ribitol-5P	231.0	97.0	11.7	13.0 ± 1.5	18.0
	231.0	79.0	11.7	13.0 ± 1.5	50.0
Ribose-1P	229.0	211.0	13.9	12.0 ± 3.3	7.5
	229.0	139.0	13.9	12.0 ± 3.3	10.3
	229.0	97.0	13.9	12.0 ± 3.3	9.2

	229.0	79.0	13.9	12.0 ± 3.3	40.0
Ribose-5P	229.0	169.0	10.4	9.8 ± 1.5	4.0
	229.0	139.0	10.4	12.0 ± 3.3	10.3
	229.0	97.0	10.4	12.0 ± 3.3	9.2
	229.0	79.0	10.4	12.0 ± 3.3	40.0
Ribulose-5P	229.0	139.0	13.3	12.0 ± 3.3	10.3
	229.0	97.0	13.3	12.0 ± 3.3	9.2
	229.0	79.0	13.3	12.0 ± 3.3	40.0
Sedoheptulose-7P	289.0	199.0	11.2	11.3 ± 1.5	10.0
	289.0	139.0	11.2	11.3 ± 1.5	20.0
	289.0	97.0	11.2	11.3 ± 1.5	19.0
	289.0	79.0	11.2	11.3 ± 1.5	57.0
Xylulose-5P	229.0	139.0	13.1	12.0 ± 3.3	10.3
	229.0	97.0	13.1	12.0 ± 3.3	9.2
	229.0	79.0	13.1	12.0 ± 3.3	40.0

Dataset S1. Overview of polar metabolite data (TBA method) from HEK293T WT, NPL KO, and NPL OE cells in control and supplemented conditions. Normalized peak areas from HEK293T cell models after 24 h in culture medium without supplementation (control) or with supplementation of 1 mM Neu5Ac, Neu5Gc, KDN, or KDO. Data are represented as the mean of three biological replicates.

Dataset S2. Overview of nucleotide sugar metabolite data (TEA method) from HEK293T WT, NPL KO, and NPL OE cells in control and supplemented conditions. Normalized peak areas from HEK293T cell models after 24 h in culture medium without supplementation (control) or with supplementation of 1 mM Neu5Ac, Neu5Gc, KDN, or KDO. Data are represented as the mean of three biological replicates.

Dataset S3. Overview of polar metabolite data (TBA method) from HEK293T WT and NPL OE cells over time in control and Neu5Ac supplement. Normalized peak areas from HEK293T cell models after 0, 2, 4, 6, and 24 h in culture medium without supplementation (control) or with supplementation of 1 mM Neu5Ac. Data are represented as the mean of three biological replicates.

Dataset S4. Overview of nucleotide sugar metabolite data (TEA method) from HEK293T WT and NPL OE cells over time in control and Neu5Ac supplement. Normalized peak areas from HEK293T cell models after 0, 2, 4, 6, and 24 h in culture medium without supplementation (control) or with supplementation of 1 mM Neu5Ac. Data are represented as the mean of three biological replicates.

Supplementary references

Gilormini PA, Lion C, Noel M, Krzewinski-Recchi MA, Harduin-Lepers A, Guerardel Y, Biot C. 2016. Improved workflow for the efficient preparation of ready to use CMP-activated sialic acids. Glycobiology 26(11):1151-1156.

Kentache T, Thabault L, Deumer G, Haufroid V, Frederick R, Linster CL, Peracchi A, Veiga-da-Cunha M, Bommer GT, Van Schaftingen E. 2021. The metalloprotein YhcH is an anomerase providing *N*-acetylneuraminate aldolase with the open form of its substrate. J Biol Chem 296:100699.

Kitagawa M, Ara T, Arifuzzaman M, Ioka-Nakamichi T, Inamoto E, Toyonaga H, Mori H. 2005. Complete set of ORF clones of Escherichia coli ASKA library (a complete set of *E. coli* K-12 ORF archive): unique resources for biological research. DNA Res 12(5):291-9.

Livak KJ, Schmittgen TD. 2001. Analysis of relative gene expression data using real-time quantitative PCR and the 2(-Delta Delta C(T)) Method. Methods 25(4):402-8.

Rahm M, Kwast H, Wessels H, Noga MJ, Lefeber DJ. 2024. Mixed-phase weak anion-exchange/reversed-phase LC-MS/MS for analysis of nucleotide sugars in human fibroblasts. Anal Bioanal Chem 416(15):3595-3604.

Sugai T, Lin CH, Shen GJ, Wong CH. 1995. CMP-KDO synthetase: overproduction and application to the synthesis of CMP-KDO and analogs. Bioorg Med Chem 3(3):313-20.

van Scherpenzeel M, Conte F, Bull C, Ashikov A, Hermans E, Willems A, van Tol W, Kragt E, Noga M, Moret EE et al. . 2022. Dynamic tracing of sugar metabolism reveals the mechanisms of action of synthetic sugar analogs. Glycobiology 32(3):239-250.
